# Phytoconstituents of *Citrus limon* (Lemon) as Potential Inhibitors Against Multi Targets of SARS‐CoV‐2 by Use of Molecular Modelling and *In Vitro* Determination Approaches

**DOI:** 10.1002/open.202300198

**Published:** 2024-06-21

**Authors:** Kannan Raman, Rajagopal Kalirajan, Fahadul Islam, Srikanth Jupudi, Divakar Selvaraj, Gomathi Swaminathan, Laliteshwar Pratap Singh, Ritesh Rana, Shopnil Akash, Md. Rezaul Islam, Firzan Nainu, Talha Bin Emran, Turki M. Dawoud, Mohammed Bourhia, Musaab Dauelbait, Rashu Barua

**Affiliations:** ^1^ Department of Pharmaceutical Chemistry JSS College of Pharmacy JSS Academy of Higher Education & Research Ooty 643001 The Nilgiris, Tamilnadu India; ^2^ Department of Pharmacy Faculty of Allied Health Sciences Daffodil International University Dhaka 1207 Bangladesh; ^3^ Narayan Institute of Pharmacy Gopal Narayan Singh University Jamuhar Sasaram, Rohtas 821305 Bihar India; ^4^ Department of Pharmaceutical Sciences (Pharmaceutics) Himachal Institute of Pharmaceutical Education and Research (HIPER) Bela, Nadaun Hamirpur, Himachal Pradesh 177042 India; ^5^ Department of Pharmacy Faculty of Pharmacy Hasanuddin University Makassar 90245 Indonesia; ^6^ Department of Pharmacy BGC Trust University Bangladesh Chittagong 4381 Bangladesh; ^7^ Department of Botany and Microbiology College of Science King Saud University P. O. BOX 2455 Riyadh 11451 Saudi Arabia; ^8^ Department of Chemistry and Biochemistry Faculty of Medicine and Pharmacy Ibn Zohr University Laayoune 70000 Morocco; ^9^ Department of Scientific Translation Faculty of Translation University of Bahri Khartoum 11111 Sudan; ^10^ Foundations of Medicine Diabetes and Obesity Research Center New York University Grossman Long Island School of Medicine 101 Mineola Blvd Mineola New York, USA

**Keywords:** Main protease, Spike protein, RdRp, MMGBSA, MD simulation, *in* anti-SARS-CoV-2 determination

## Abstract

In the present work, phytoconstituents from *Citrus limon* are computationally tested against SARS‐CoV‐2 target protein such as Mpro ‐ (5R82.*pdb*), Spike ‐ (6YZ5.*pdb*) &RdRp ‐ (7BTF.*pdb*) for COVID‐19. Docking was done by glide model, QikProp was performed by *in silico* ADMET screening & Prime MM‐GB/SA modules were used to define binding energy. When compared with approved COVID‐19 drugs such as Remdesivir, Ritonavir, Lopinavir, and Hydroxychloroquine, plant‐based constituents such as Quercetin, Rutoside, Naringin, Eriocitrin, and Hesperidin. bind with significant G‐scores to the active SARS‐CoV‐2 place. The constituents Rutoside and Eriocitrin were studied in each MD simulation in 100 ns against 3 proteins 5R82.*pdb*, 6YZ5.*pdb* and 7BTF.*pdb*.We performed an assay with significant natural compounds from contacts and *in silico* results (Rutin, Eriocitrin, Naringin, Hesperidin) using 3CL protease assay kit (B.11529 Omicron variant). This kit contained 3CL inhibitor GC376 as Control. The IC_50_ value of the test compound was found to be Rutin −17.50 μM, Eriocitrin−37.91 μM, Naringin−39.58 μM, Hesperidine−140.20 μM, the standard inhibitory concentration of GC376 was 38.64 μM. The phytoconstituents showed important interactions with SARS‐CoV‐2 targets, and potential modifications could be beneficial for future development.

## Graphical abstract

This research has conducted a combine experiment both *in vitro*, and computational analysis to investigate the potential efficacy of phytoconstituents present in *Citrus limon* (lemon) against SARS‐CoV‐2. Overall analysis has reported that a significant binding affinity against the targeted proteins which is shown by phytoconstituents such as Rutin, Eryocitrin, Naringin, and Hesperidine, indicating that these compounds may be useful treatments option against COVID‐19 and immunomodulators. However, further clinical research is need in broad scale to confirm these finding.

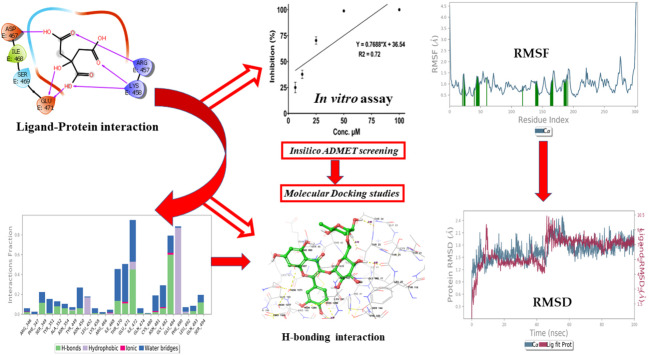

## 1 Introduction

Coronavirus disease (COVID‐19) is mainly caused by coronaviruses (CoVs) family belonging to the Middle East respiratory syndrome (MERS), & severe acute respiratory syndrome (SARS) virus which contains one positive‐strand RNA.[^1^, ^2^] China was the first to hit, and it swiftly spread to more than 200 other countries.[^3^, ^4^, ^5^] From the WHO data, as of June 2022, COVID‐19 has affected about 550 million people worldwide, with around 6.3 million persons dying as a result. Most of the COVID‐19 affected patients are mainly more asymptomatic,[^3^, ^4^] understanding how SARS‐CoV, MERS‐CoV, and SARS‐CoV‐2 are transmitted and how to inactivate the CoVs are vital concerns.[^5^, ^6^, ^7^, ^8^, ^9^] Additionally, CoVs spread more quickly when it was passed from person to person. It is tough to identify some fresh treatments with all clinical trials, determination of unwanted consequences. because COVID‐19 optimistic cases are increasing more aggressively day per day. Vaccinations have been reported, however, their effectiveness against COVID‐19 is unknown. Therefore, the drug repurposing or identification of natural compounds is widely accepted and an effective strategy in identifying novel medication for the effective treatment of COVID‐19 via *in silico* drug screening experiments.[^10^, ^11^]

As an essential component of Indian traditions, the Indian medical system is as old as Indian history itself. Plants are used as common medications and the majority of the general public in the form of Ayurveda, Siddha, Unani, and so on, still receives medical care through Indian traditional medicinal systems despite the success of allopathic drugs. The majority of the drugs used in the formulations of Ayurveda are derived from plant sources, which are inexpensive and have minimum negative consequences. The *Citrus limon* is belonged to the Rutaceae family, it is grown in different nations throughout the world and is generally known as lemon fruit. Supported by numeric scientific studies. Because of its high vitamin content, lemon juice is a traditional food or herbal cure for the treatment of common cold and flu.^[12]^ It is a rich source of bioactive molecules having plethoric biological properties were reported as anti‐cancer activity, antioxidant effects, anti‐inflammatory,[^13^, ^14^, ^15^] antimicrobial,[^16^, ^17^, ^18^] anti‐parasitic effects,^[19]^ anti‐allergic effects,^[20]^ anti‐diabetic effects,[^21^, ^22^] and anti‐obesity effects.^[23]^ Flavonoids such as eriocitrin, neoeriocitrin, vitexin,eriodictoyl,hespiridin, naringin, rutoside, diosmetin, diosmin, luteolin, orientin, quercetin, apigenin, etc. are found in the plant *C. limon* along other chemical constituents such as with vitamin C, scopoletin, dihydroferulic acid, citric acid, quinic acid, and so on.^[17]^ Lemon juice is commonly used to treat scurvy, sore throats, fevers, rheumatism, high blood pressure, and chest pain, and it is high in vitamin C, which aids in infection prevention.^[24]^

As part of our ongoing investigation into novel heterocyclic molecules, we have created and tested different heterocyclic compounds for biological activity using *in silico* approaches, and wet lab method. Various modules of Schrödinger suite LLC like such as Glide, Qikprop, and Prime were employed to predict the binding affinity between protein and ligand, calculating relative binding energy, toxicity profiling, and so on. These *in silico* tools aid in exploring the therapeutic potential of natural compounds against COVID‐19. This study highlights to evaluate the binding pattern of phytoconstituents found in *C. limon* through molecular docking and molecular dynamics approach. Also, these studies will pave with key findings regarding the pharmacophoric features responsible for designing potential drugs.

## 2 Materials and Methods

### 2.1 Molecular Docking Screening

The SARS‐CoV‐2 Mpro receptor 3‐D crystal structure (*pdb* ID: 5R82, Resolution: 1.31 Å), SARS‐CoV‐2 spike glycoprotein (*pdb* ID: 6YZ5, Resolution: 1.80 Å) & SARS‐CoV‐2 RdRp (*pdb* ID: 7BTF, Resolution: 2.95 Å) were collected from the protein data bank. Proteins were created using the Schrödinger suite‘s wizard. via removing equivalent binding sites., water molecule beyond 5 Å, and refining bond orders protein was reduced using the OPLS3e molecular force field, and the grid box built to identify the active site‘s centroid. The Prime module of the Schrödinger Suite was utilized to add the missing chains and loops.[^25^, ^26^, ^27^, ^28^, ^29^, ^30^, ^31^]

The LigPrep module of the Schrodinger suite has been used to produce phytoconstituents (L1‐L15) from the *C. limon* plant. The OPLS‐3e force field decreased the energy of the ligand and optimized for their geometry. Ionization and tautomeric states between pH values of 6.8 and 7.2 were produced using the Epik module. Each ligand had a low energy conformation created for it, and the docking studies used the optimized ligands. The Prepared ligand was docked into the binding pocket of COVID–19 targets such as the Mpro, spike glycoprotein and RdRp by the XP (Extra precision) mode of the Glide module of the Schrödinger suite. The most Glide‐rated binding method was selected. These scores highlight, and penalize favorable H‐bonds, metal‐ligand interactions, and lipophilic interactions. The docking data were analysed using the Glide module XP visualizer.^[32]^ The Glide scores were compared with standard compounds which are recommended for COVID‐19 drugs such as Remdesivir, Ritonavir, Lopinavir, and Hydroxychloroquine.[^33^, ^34^] The Glide scoring functioncombines parameters such as lipophilic perseverance, electrostatic forces, and hydrogen bonding.

### 2.2 In Silico ADMET Screening

The *in silico* ADMET properties of the proposed ligands **L1‐L15** were analyzed by the Qikprop module of Schrodinger suite. Physicochemical properties like molecular mass, dipole moment, number of H‐bond donors and acceptors, logP value, violence in the rule of 5, oral absorption, etc. were mentioned in Supplementary Table S1.^[35]^

### 2.3 Binding Free Energy Calculation by Using the Prime/MM‐GBSA Approach

A post‐docking energy minimization was performed. The Prime module Schrödinger suite was utilized to calculate the Molecular Mechanics‐Generalized Born Surface Area (MM‐GB/SA) energy for the ligand‐receptor complex. The complexes were energy minimized usingOPLS3e force field with generalized born continuum VSGB 2.0 implicit solvent model.[^36^, ^37^]

### 2.4 Molecular Dynamics (MD) Simulation

The stability and the dynamic changes for the docked poses of **L14_rutoside and L12_eriocitrin in complex with** SARS‐CoV‐2 Mpro receptor, spike glycoprotein, and RdRp were determined by performing a 100 ns MD simulation. Orthorhombic periodic boundary conditions were used to solve the molecule using crystallographic TIP3P waters for the ten buffer regions. The overlapping water molecules were eliminated, and the solution was then neutralized by the addition of Na^+^ and Cl^−^ as counter ions. Energy minimization was carried out in the Desmond module of the Schrödinger Suite using the OPLS3e force field. The systems were kept at a constant temperature (300 K) and pressure (1 bar) via utilising a Nose‐Hoover thermostat and barostat ensemble (NPT).[^38^, ^39^] Following the application of conjugate gradient techniques, a hybrid energy reduction strategy with 1000 steps of steepest descent was adopted.For memory restriction and energy minimization, the Broyden‐Fletcher‐Goldfarb‐Shanno (LBFGS) algorithm with a gradient of 1 kcal/mol was used. Short‐range van der Waals and Coulomb interactions were calculated using a Smooth Particle Mesh Ewald method with a cut‐off radius of 9.^[40]^ The dynamics of bonded, close, and far‐bonded interactions were studied using multiple time‐step RESPA integrations (reference system propagator methods) with 2 fs, 2 fs, and 6 fs, respectively. Data was gathered every 100 ps, and Maestro graphical interphase was used to analyze the obtained trajectory. The results were interpreted to understand the RMSD, RMSF, interaction fraction of ligand‐protein contacts and the interacting hydrogen bonds.^[41]^

### 2.5 In Vitro Assay: The 3CL Protease or Main Protease (Mpro) (B.1.1.529, Omicron Variant, P132H Mutant) SARS‐CoV‐2 Assay Kit

#### 2.5.1 Assay Protocol

All samples and controls must be tested two times. Make as much DTT‐containing buffer as needed for the experiment. Warm up the 3CL protease keep (B.1.529, Omicron mutantSARS‐CoV‐2) on ice before use. Rotate the tube containing the enzyme briefly to recover the entire contents of the tube. In Assay Buffer, dilute the 3CL protease (B.1.529, Omicron Mutant) to 1.5 ng/l (45 ng per response). 30 l of diluted protease in wells labeled “Positive Control,” “Inhibitor Control,” and “Test Inhibitor” Aliquot and store the leftover solution at −80 °C.Preincubate at room temperature for 30 minutes with slow shaking. Diluent Solution (no inhibitor): To the “Blank” and “Positive Control” wells, add 10 μl assay buffer. The final DMSO concentration in the experiment should not be more than 1 %. Dilute 25 l of 3CL protease substrate (10 mM) in 1 ml of DTT‐containing assay buffer The plate should be incubated for 4 hours at room temperature following plate closure. Measure the fluorescence intensity using a microtiter plate reading fluorimeter that can excite at 360 nm and detect emission at 460 nm. The intensity of fluorescence can also be monitored kinetically. All other values are subtracted from the “Blank” value.[^42^, ^43^]

## 3 Results and Discussion

**Figure 1:**
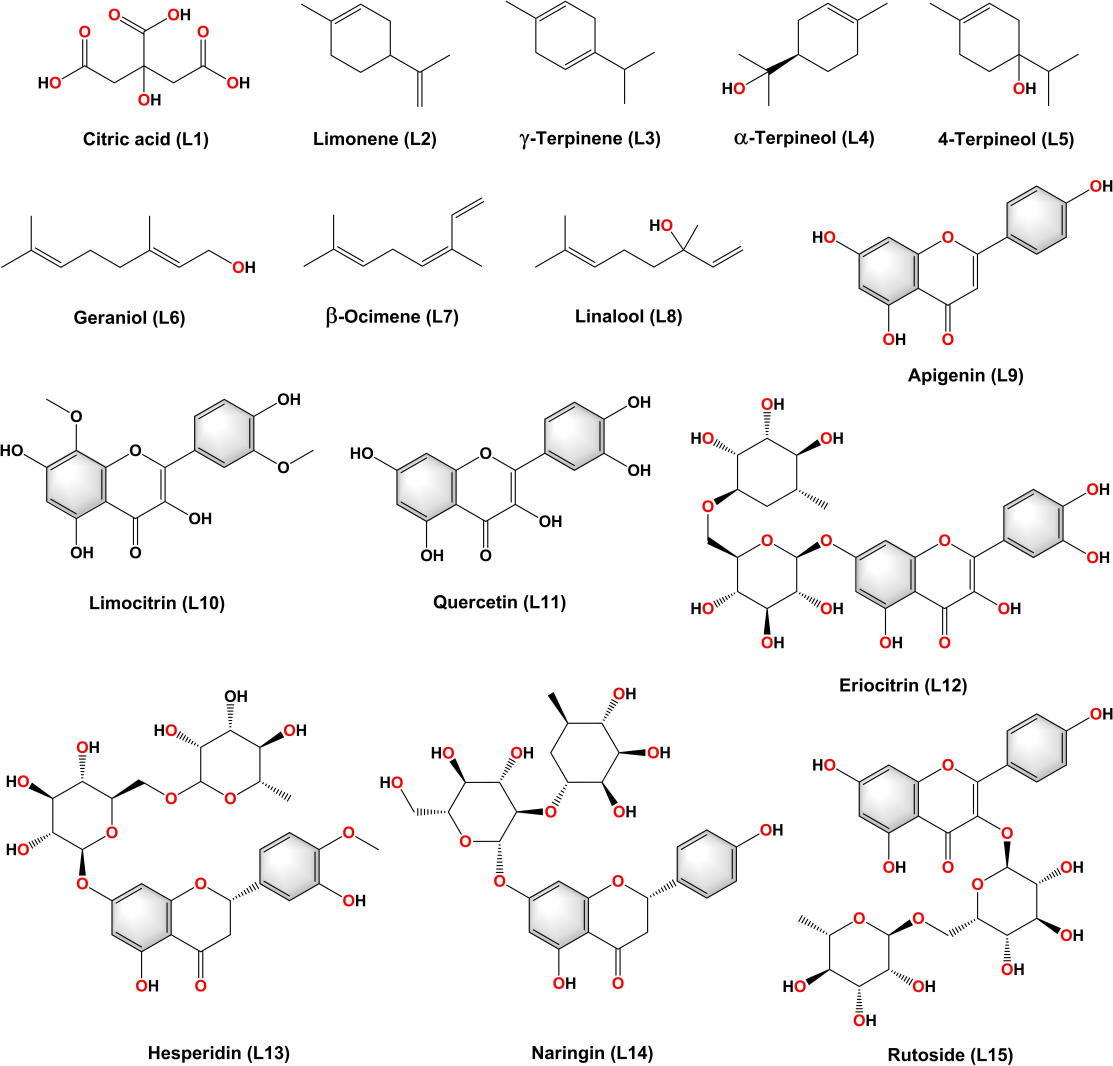
Structures of major Phytochemical constituents of *Citrus limon*.

**Figure 2:**
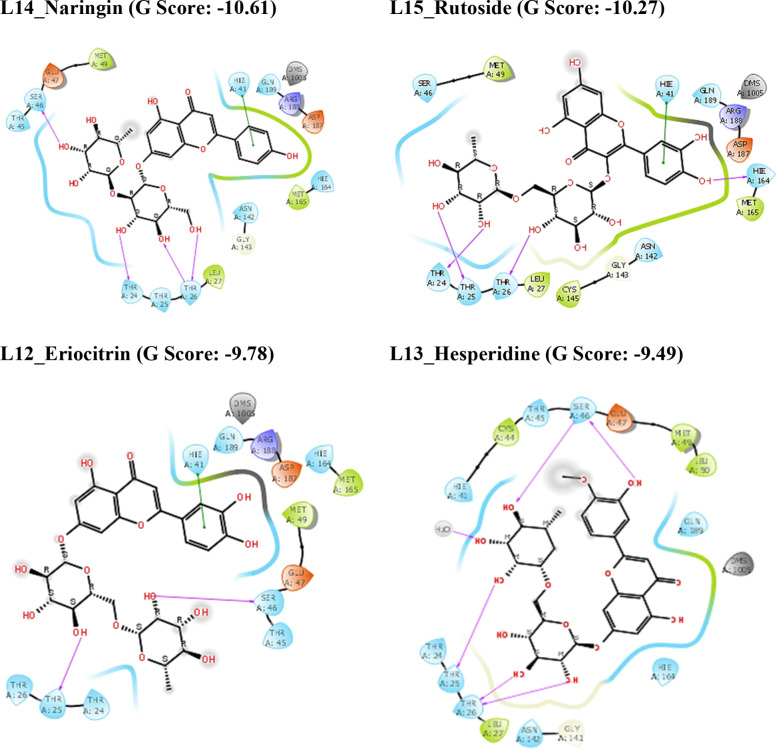
2D ligand interaction diagrams of compounds in the catalytic pockets of SARS‐CoV‐2 Mpro (G score in kcal/mol).

The chemical structures of major phytochemical constituents from *C. limon* were shown in Figure 1. Results of molecular docking and MM‐GBSA methods against three targets were summarized in Table 1 and 2 respectively and the 2D interaction diagrams and 3D interaction images were illustrated in Figures 2 and 4 and Figure 5 respectively. The results showed that the chemical nature of the substituents had a large impact on the SARS‐CoV‐2 inhibitory property of the compounds L1‐L15.[^44^, ^45^, ^46^]

**Table 1:** Docking studies for phytochemical constituents of *Citrus limon* with SARS‐CoV‐2, Mpro, Spike protein, and RdRp.

Compound	Main protease (5R82)			Spike protein (6YZ5)			RdRp (7BTF)		
	Glide score	Lipophilic EvdW	H Bond	Glide score	Lipophilic EvdW	H Bond	Glide score	Lipophilic EvdW	H Bond
L15_Rutoside	**−10.27**	−4.14	−5.43	**−7.96**	−1.79	−6.1	**−11.73**	−2.85	−7.35
L14_Naringin	**−10.61**	−4.56	−4.96	**−3.02**	−1.98	−1.92	**−10.45**	−3.13	−5.95
L12_Eriocitrin	**−9.78**	−4.32	−5.02	**−6.9**	−2.18	−5	**−11.03**	−2.76	−6.68
L13_Hesperidine	**−9.49**	−3.89	−5.15	**−5.44**	−2.47	−3.44	**−9.51**	−2.92	−5.79
L10_Limocitrin	**−8.06**	−3.25	−3.59	**−3.89**	−1.85	−2.43	**−6.31**	−2.91	−2.55
L11_Quercetin	**−7.41**	−4.08	−2.4	**−5.36**	−1.07	−3.03	**−7.02**	−3.08	−3.18
L9_Apigenin	**−6.02**	−4.11	−1.14	**−2.16**	−1.09	−1.13	**−5.12**	−2.72	−1.56
L1_Citric acid	**−5.5**	−0.89	−3.7	**−5.91**	−0.4	−4.24	**−5.53**	−1.24	−2.97
L5_4_Terpeneol	**−4.7**	−3.04	−0.76	**−2**	−0.69	−0.7	**−3.02**	−2.26	−0.24
L4_ α Terpeneol	**−4.55**	−3.06	−0.94	**−2.31**	−1.09	−0.7	**−3.31**	−2.11	−0.7
L3_Terpinene	**−4.17**	−3.65	0	**−1.39**	−1.07	0	**−2.77**	−2.16	0
L6_Geraniol	**−3.85**	−3.53	−0.7	**−1.95**	−0.8	−1.05	**−3.07**	−1.93	−1.05
L2_Limonene	**−3.68**	−3.52	0	**−1.1**	−1.26	0	**−2.8**	−2.57	0
L8_Linalool	**−3.48**	−3.03	−0.7	**−1.63**	−1.28	−0.7	**−2.97**	−1.96	−1.03
L7_β Ocimene	**−3.07**	−3.57	0	**−0.58**	−1.34	0	**−2.45**	−2.96	0
Remdesivir(std)	**−6.38**	−4.57	−1.77	**−2.63**	−2.61	−1.14	**−6.47**	−4.81	−2.13
Ritonavir(std)	**−7.48**	−6.57	−1.7	**−3.31**	−1.75	−1.51	**−5.06**	−5.87	−0.96
Lopinavir(std)	**−6.94**	−6.01	−1.12	**−1.59**	−1.79	−1.36	**−3.88**	−3.86	−1.11
Hydroxychloroquine(std)	**−5.47**	−3.15	−1.75	**−1.82**	−1.85	−0.89	**−5.43**	−3.28	−1.6

Std: Standard

**Table 2:** Free energy Binding calculation & PrimeMM‐GBSA approach.

Compound	Main protease (5R82)			Spike protein (6YZ5)			RdRp (7BTF)		
	Δ_Bind_	Δ_Coul_	Δ_Vdw_	Δ_Bind_	Δ_Coul_	Δ_vdW_	Δ_Bind_	Δ_Coul_	Δ_Vdw_
L15_Rutoside	−62.04	−41.82	−32.02	−35.63	−35.62	−32.21	−45.81	−35.11	−36.02
L14_Naringin	−45.01	−35.18	−34.66	−17.67	−17.67	−4.27	−33.88	−23.67	−33.63
L12_Eriocitrin	−53.65	−11.83	−32.48	−47.82	−47.82	−24.48	−21.87	−32.12	−37.48
L13_Hesperidine	−50.25	−51.94	−48.81	−16.84	−16.84	−30.64	−41.91	−28.68	−42.81
L10_Limocitrin	−41.38	−24.75	−36.09	5.51	5.51	−19.87	−28.65	−29.67	−32.09
L11_Quercetin	−39.17	−18.53	−35.40	−49.98	−49.98	−8.47	−20.53	−24.43	−39.42
L9_Apigenin	−33.67	−7.80	−35.62	−20.23	−20.23	−17.53	−11.82	−18.23	−31.65
L1_Citric acid	−44.94	−60.81	−9.48	−22.98	−22.98	−6.75	−32.84	−26.70	−12.42
L5_4_Terpeneol	−11.76	13.55	−16.41	−5.53	20.13	−12.78	−14.55	−21,32	−19.61
L4_ α Terpeneol	−12.91	−17.71	−10.47	−23.77	−24.20	−15.18	−16.73	−20.36	−16.43
L3_Terpinene	−23.58	0.50	−17.85	−4.80	22.20	−17.91	−10.51	−19.37	−14.83
L6_Geraniol	−6.46	0.62	−15.76	−11.80	11.67	−10.65	−5.12	−21.89	−17.72
L2_Limonene	−20.97	30.26	−32.46	−16.06	11.67	−25.05	−20.23	−15.06	−36.76
L8_Linalool	−27.34	−0.84	−25.03	−29.67	−12.23	−15.84	−8.84	−9.43	−15.09
L7_β Ocimene	−6.87	14.27	−18.86	−21.02	−6.11	−10.30	1.23	2.46	−16.81

### 3.1 Molecular Docking Studies

The chemical constituents of *C. limon* were docked towards the binding pockets of SARS‐CoV‐2 Mpro, Spike protein, and RdRp to ascertain their binding modes.[^47^, ^48^] As shown in Table 1, it is shown that the flavonoids present in the lemon such as Rutoside, Naringin, Eriocitrin, Hesperidin, and Quercetin have more significant G‐score against all three targets when compared to standard compounds. Because of their strong hydrogen bonding interactions, the aforementioned chemicals have a high affinity for binding to receptors.

From the docking results, the **L1‐L15** compounds exhibited a similar mode of interactions with the binding pocket residues of SARS‐CoV‐2 Mpro between Thr24 andGln189, with SARS‐CoV‐2 spike glycoproteinbinding siteresidues between Arg454 and Phe490 and with RdRpfor the residues between Leu172 and Pro677. The results were summarized in Table 1 and the docking poses of all compounds with targets are shown in the supplementary Figures S1a–c.

**Figure 3:**
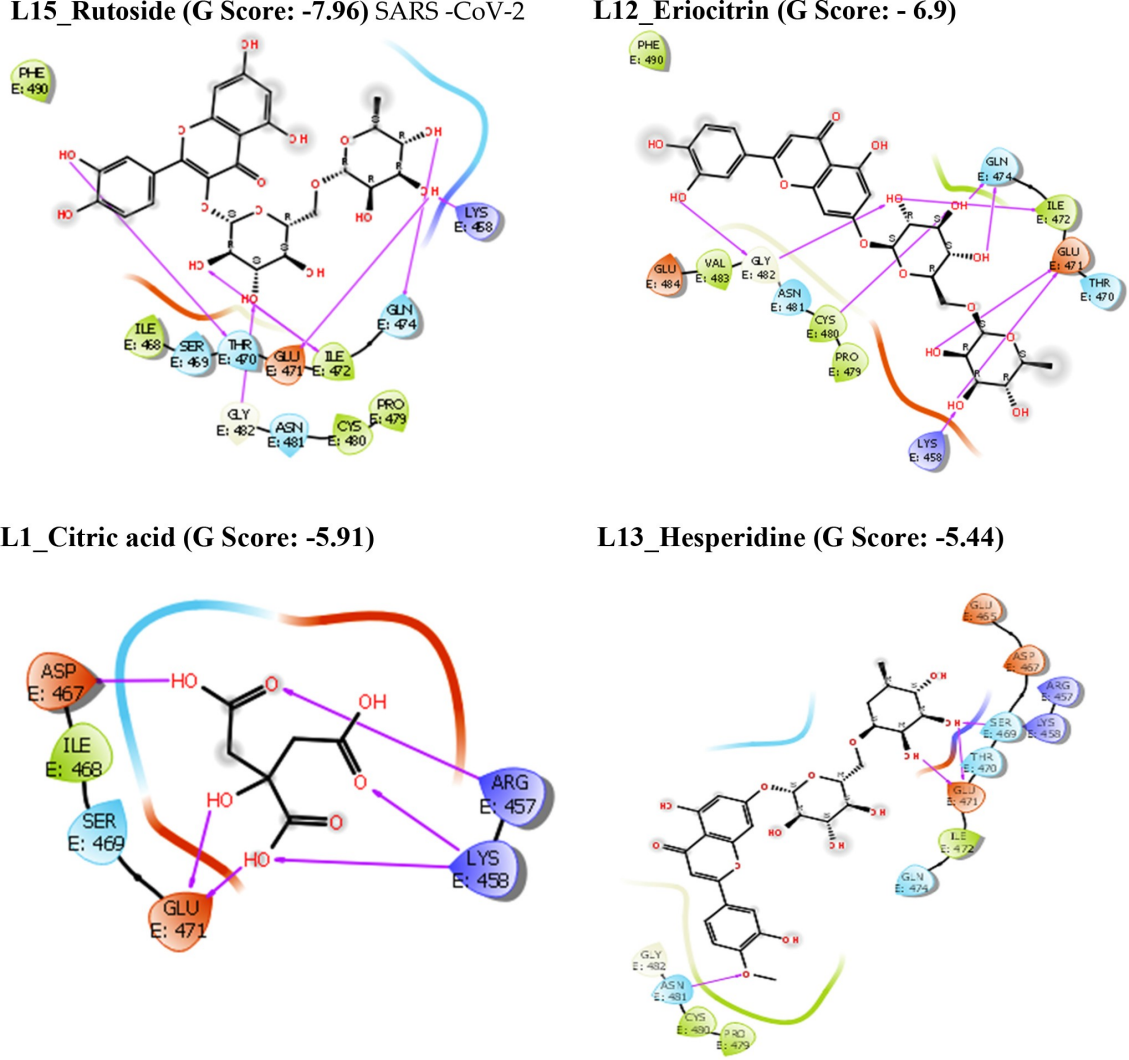
2D ligand interaction diagrams of compounds in the catalytic pockets of SARS‐CoV‐2 Spike protein (G score in kcal/mol).

**Figure 4:**
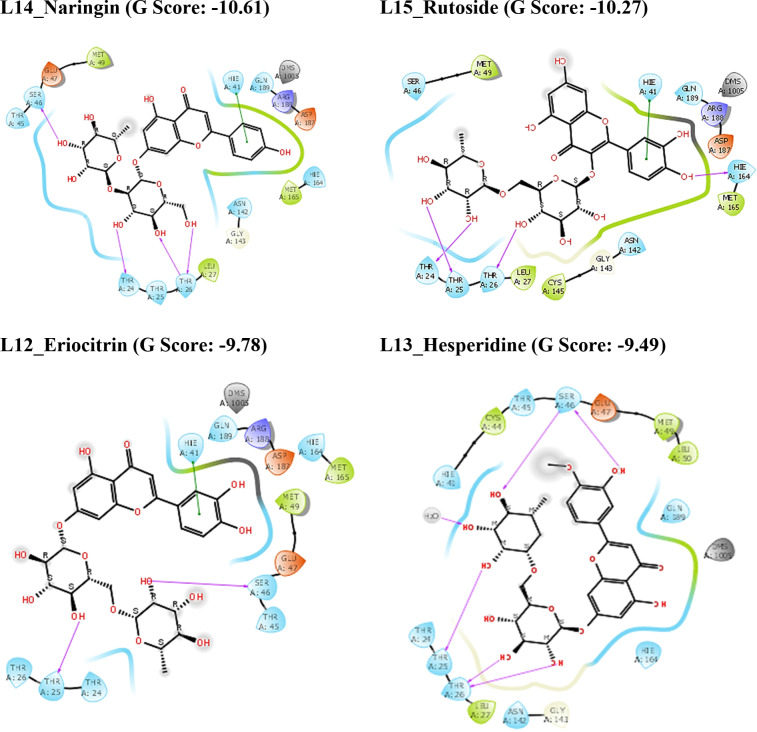
2D ligand interaction diagrams of compounds in the catalytic pockets of SARS‐CoV‐2 RdRp(G score in kcal/mol).

The compound L15_Rutoside has a significant G‐score with all three targets such as Mpro, spike protein, and RdRp with −10.27, −7.96, and −11.73 kcal/mol respectively. L12_Eriocitrin has a significant G‐score with all three targets with −9.78, −6.9, and −11.03 kcal/mol respectively. Similarly, the compound L13_Hesperidine has a significant G‐score with all three targets with −9.49, −5.44, and −9.51 kcal/mol respectively. But the compound L14_Naringin has a significant G‐score with Mpro and RdRp with −10.61 and −10.45 kcal/mol respectively. The other compounds L10_Limocitrin and L11_Quercetin also have significant G‐score with all three targets. The compound L1_Citric acid has had a significant G‐score with spike protein. The G‐score of all the above flavonoids present in the lemon is good mainly due to the hydrogen bond interactions of hydroxyl groups with amino acids. From the *in silico* results, binding affinities of flavonoids present in the lemon are more significant when compared with the standard drugs against all three targets of SARS‐CoV‐2. The 2D interactions of significantly active compounds with good G‐scores are shown in Figures 2, 3, 4.

#### 3.1.1 CoV‐2 RdRp (7BTF.*pdb*)

**Figure 5:**
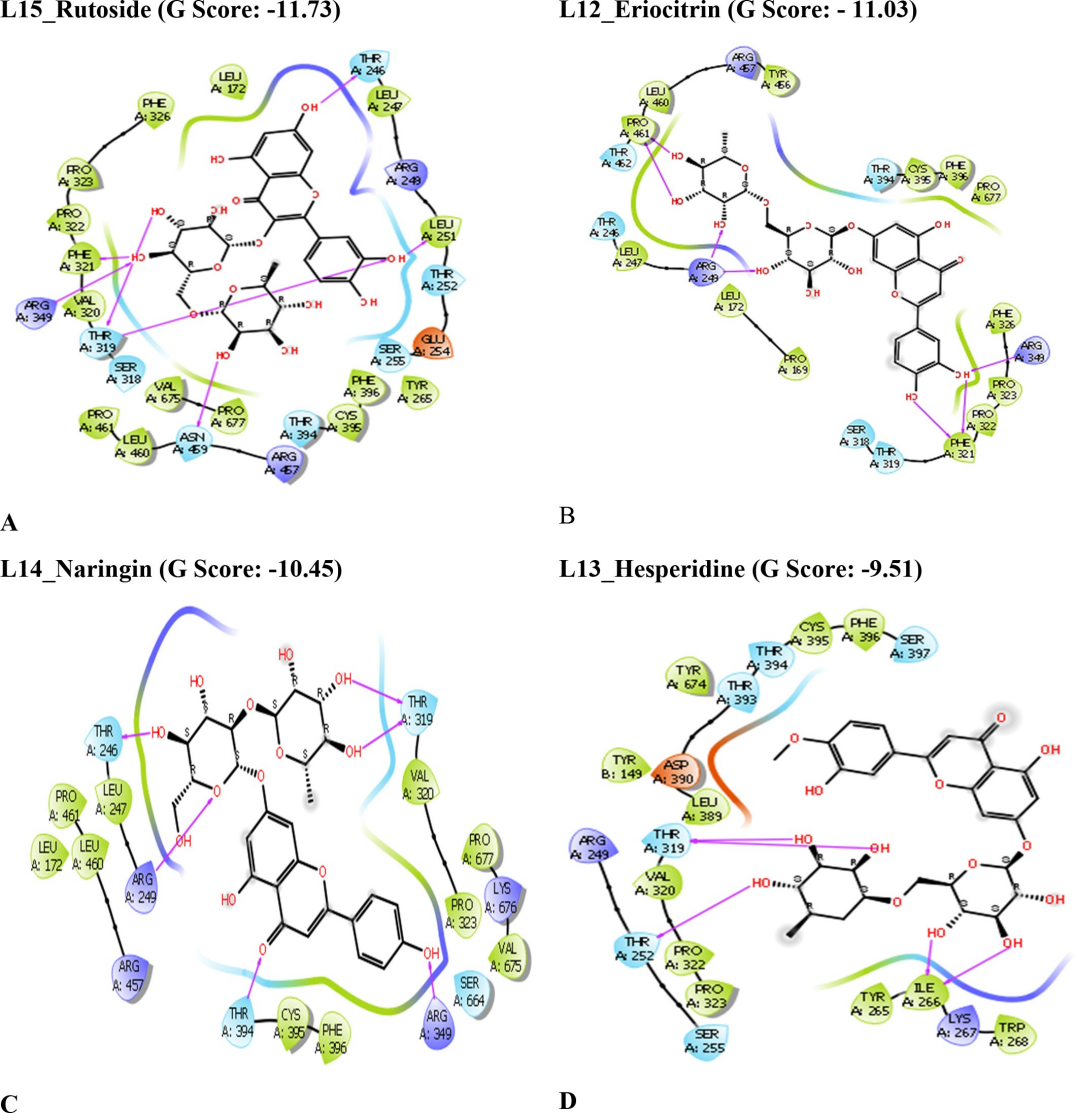
H‐bonding interaction of L15_Rutoside with (a) SARS‐CoV‐2 Mpro, (b) SARS‐CoV‐2 Spike protein and (c) SARS‐CoV‐2 RdRp.

It was discovered from the glide XP binding mechanisms that the ligands mainly build hydrogen bonding interactions with various amino acid residues surrounding the binding pocket. The top‐scored most significantly active compound L15_Rutoside formed exhibited more no. of hydrogen bonding interaction with all three targets. The ligand L15_Rutoside exhibited hydrogen bonding interaction withThr24 (H‐bond length: 2.08 Å), Thr25 (H‐bond length: 2.13 Å), Thr26 (H‐bond length: 2.26 Å), and His164 (H‐bond length: 2.77 Å) residues with a Mpro target. The ligand L15_Rutoside exhibited hydrogen bonding interaction with Thr470 (H‐bond length: 2.01 Å), Ile472 (H‐bond length: 1.73 Å), Gly474 (H‐bond length: 1.96 Å), Gly482 (H‐bond length: 2.15 Å), and Lys468 (H‐bond length: 1.95 Å) residues with the spike protein target. Similarly, the ligand L15_Rutoside exhibited hydrogen bonding interaction with Thr246 (H‐bond length: 1.90 Å), Leu251 (H‐Bond length: 2.04 Å), Thr319 (H‐Bond length: 2.52 Å), Phe321 (H‐Bond length: 1.96 Å), Arg349 (H‐Bond length: 2.11 Å) and Asn469 (H‐Bond length: 2.43 Å) residues with RdRp protein target. The Hydrogen bond interactions were shown in Figures 5a–d respectively.

### 3.2 In Silico ADMET Screening

The *in silico* ADMET screening for the molecules can be predicted by using Qikprop module of Schrödinger suite. *In silico* technique can be used to examine the ADMET properties for the molecules L1‐L15. The compounds range in molecular weight from 154 to 610 g.mol^−1^. The compounds′ dipole moments range from 0.2 to 7.5. The chemicals′ estimated hydrogen bond donors range from 0 to 9. The number of compounds′ hydrogen bonding acceptors is thought to be between 0–20. The number of the chemicals′ probable metabolites is in the range of 3–10. There have been 0–3 infractions of Lipinski's rule of 5. In the supplementary table S1, molar volume, pKa values, Vander Walls volume, and several chiral centers are shown. Most of the ADMET characteristics are within the recommended ranges, according to the results. However, for some hit compounds, the ADMET properties are not within the recommended ranges, which will be improved by group modifications or by employing other formulations.[^49^, ^50^, ^51^]

### 3.3 Binding Free Energy Calculation by Using the Prime MM‐GBSA Methods

For the SARS‐CoV‐2 Mpro, Spike protein, and RdRp, the stability of docking was evaluated using the MM‐GBSA free binding energy, and the results are displayed in Table 2. Prime MM‐GBSA ΔG bind, the binding free energy, is calculated with the equation.[^52^, ^53^, ^54^]

(1) ΔG(bind)=E_complex(minimized)-(E_ligand(minimized)+E_receptor(minimized))

Δ _Bind:_ Total binding energy_;_ Δ _Coul:_ Coulombicenergy_;_Δ_Vdw:_ Van der Waal energy.

The post‐docking minimization studies (MMGB/SA) revealed that the selected compounds showed good binding energy (Δ_Bind_) with the catalytic residues of Mpro (−6.82 to −62.04 kcal/mol)and moderate binding with spike protein (5.51 to −49.98 kcal/mol) and RdRp (1.23 to −45.81 kcal/mol). For the selected compounds the total binding energy was favored by Van der Waal and coulombic energy terms with high negative values. From the selected compounds Rutosideexhibited good binding affinity with Mpro (−62.04 kcal/mol) and RdRp (−45.81 kcal/mol), whereas, quercetin exhibited good binding affinity with spike protein (−49.98 kcal/mol).

### 3.4 Molecular Dynamics Simulation

#### 3.4.1 Molecular Dynamics Simulation Study of Rutoside/5R82 Complex

**Figure 6:**
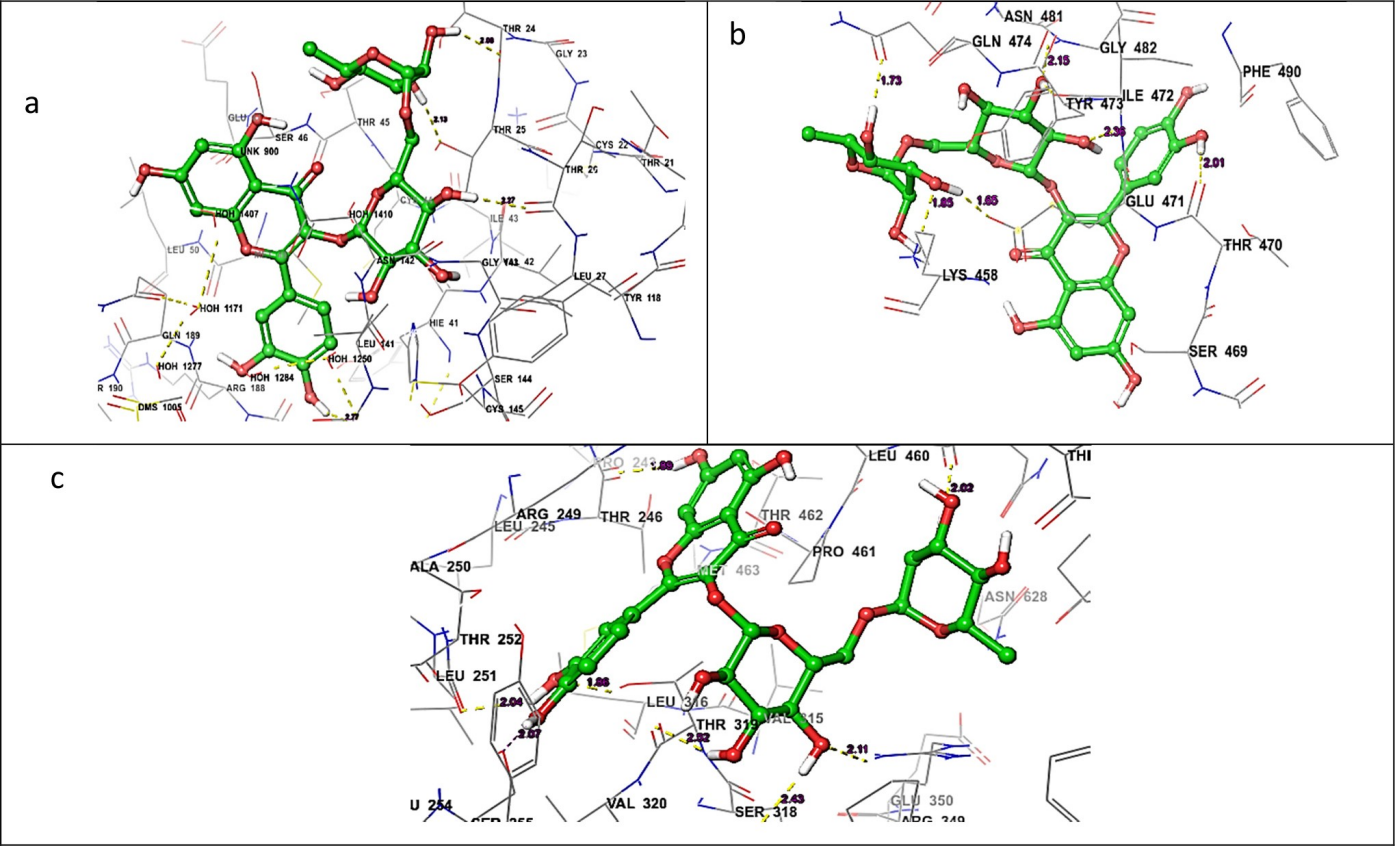
(A) RMSD (Å) of simulated protein 5R82.*pdb* in complex with inhibitor rutosideover 100 ns MD simulation (B) RMSF of simulated protein 5R82.*pdb* in complex with inhibitor rutoside over 100 ns MD simulation (C)Interaction fraction of 5R82.*pdb* complexed with rutosideover 100 ns MD simulation.

The 100 ns simulation was performed by placing docked pose of rutoside/5R82 complex in an orthorhombic box solvated with TIP3P waters and the total charge of the system was neutralized by adding 30 Na^+^ and 27 Cl^−^ ions.[^55^, ^56^] The total system consists of 33776 atoms and 9667 water molecules. Since the MD trajectory of 100 ns simulated rutoside/5R82 complex, it was emphasized that the RMSD of protein Cα atoms achieved equilibrium after 14 ns and stabilized until 49 ns with an RMSD range of 1.45 to 2.12 Å. Then the complex showed high fluctuation from 49 to 60 ns with RMSD reaching 2.48 Å, then plummeting with a stable graph of 1.5 to 2.0 Å throughout the end of the simulation (Figure 6A). From Figure 6A the ligand also observed the same fate concerning protein in RMSD fluctuations until 49 ns then a surge followed by stabilization throughout the end of the simulation. The instability in ligand RMSD was observed until 49 ns ranging from 1.4 to 10.5 Å and stabilized after 60 ns with 7.6 to 8 Å.The RMSF (root mean square fluctuation) plot (Figure 6B) showed that the protein is stable throughout the simulation, except for amino acids of the tail portion, with minimum RMSF values of 0.4 to 2.02 Å.

**Figure 7:**
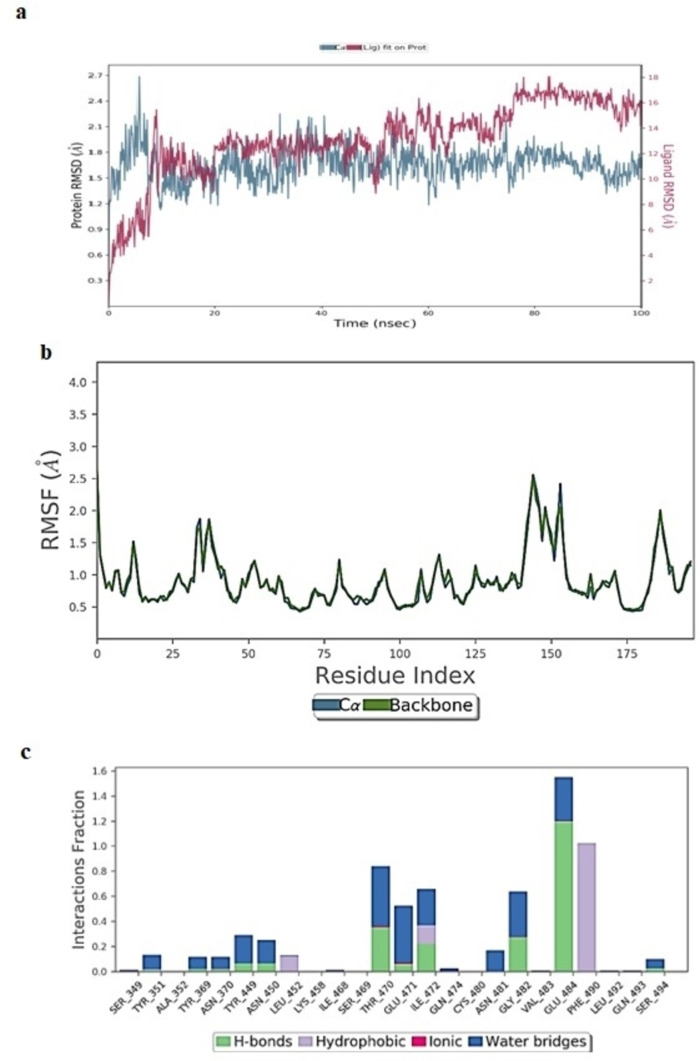
(A) RMSD (Å) of simulated protein 6YZ5.*pdb* in complex with inhibitor rutosideover 100 ns MD simulation (B) RMSF of simulated protein 6YZ5.*pdb* in complex with inhibitor rutoside over 100 ns MD simulation (C) Interaction fraction of 6YZ5.*pdb* complexed with rutosideover 100 ns MD simulation.

The monitored protein interaction fraction plot indicated that Rutoside was stabilized in the catalytic pocket by forming hydrogen bonding and water‐bridged interactions, of which Thr26 formed strong hydrogen bonding within 100 ns simulation time (Figure 6C). This may be achieved by forming multiple hydrogen bonds for longer simulation times. From Figure 7, more contacts, nearly 10–18 were observed until 49 ns which were then diminished to 6–8 contacts throughout the simulation of 100 ns.Thr26 formed very stable interactions followed by Thr24, His41, Cys44, Glu166, Glu189 forming a medium range, and Thr25, Ser46, Asn119, Asn142, Gly143, Ser144, Cys145 with low range interactions. The 3D ligand interaction diagram revealed that the two hydroxyl groups of the mannopyranosyl ring in rutoside formed two stable hydrogen bonds with a whopping 158 % of the total 100 ns MD simulation trajectory. Cys44 formed water‐bridged interaction with a hydroxyl group of mannopyranosyl moiety at 41 % of the total simulation trajectory. Intramolecular H‐ bond was observed between the fifth hydroxyl group &carbonyl oxygen at the fourth position of 5,7‐dihydroxy‐4H‐1‐benzopyran‐4‐one ring.Supplementary Figure S2 shows a representation of protein‐ligand interactions. The 2D interaction diagram Supplementary Figure S3 shows that the hydrogen bond formed by the docking pose with Cys44 is preserved in the MD trajectory pose. The Hydroxy group present in the mannopyranosyl moiety in rutoside donated one hydrogen bond to Cys44 with 41 % through the water molecule and also accepted one hydrogen bond with Thr26 with 72 % of the total simulation time. Another hydroxy group present in the mannopyranosyl moiety in Rutoside donated one hydrogen bond with Thr26 with 86 % of the total simulation time. The root means square fluctuation (Figure 10) of the ligand concerning with initial frame was observed in the range of 1.0–2.78 Å. Other ligand properties (Supplementary Figure S4) like the radius of gyration, molecular surface area (MSA), solvent accessible surface area (SASA), and polar surface area (PSA) of ligands were observed in the range of 4.35–5.17 Å, 437.15–487.45 Å^2^, 290.70–583.83 Å^2^ and 421.55–495.77 Å^2^ respectively.

#### 3.4.2 Molecular Dynamics Simulation Study of Rutoside/6YZ5 Complex

The docked pose of rutoside/6YZ5 was simulated at 100 ns simulation was performed by placing it in an orthorhombic box solvated with TIP3P waters and the system was neutralized by adding 23 Na^+^ and 24Cl^−^ ions. The total system consists of 28375 atoms and 8354 water molecules. The 100 ns MD trajectory of the simulated rutoside/6YZ5 complex revealed that the RMSD of protein Cα and backbone atoms were observed in the range of 1.75 to 2.72 Å and 1.70 to 2.75 Å respectively with a minor spike in RMSD at 85 ns (Figure 7A). From Figure fluctuations in ligand, RMSD was observed with a timescale till 75 ns which was then stabilized throughout the end of the simulation. The ligand RMSD was maintained at 14.5–17 Å from 18 to 50 ns and at 11.8–15 Å from 52 to 75 ns. After 75 ns the ligand RMSD was stabilized at 17.5 Å. The RMSF (root mean square fluctuation) plot as represented in Figure 7B indicated that the protein is stable throughout the simulation, except amino acids 145–160 having RMSF values of near to 2.50 Å.

The monitored protein interaction fraction plot indicated that rutoside was stabilized in the catalytic pocket of 6YZ5 by forming hydrogen bonds, and hydrophobic and water‐bridged interactions. Tyr449, Thr470, Glu471, Ile472, Glu‐484and Phe‐490 during 100 ns simulation time (Supplementary Figure S6). Figure 7C showed the history of interactions between proteins and ligands. The 3D ligand interaction diagram illustrated that Glu484 formed water‐bridged and stable hydrogen bonds with two hydroxyl groups of 3,4‐dihydroxy phenyl ring with a total 113 % of total 100 ns MD simulation trajectory. Phe490 formed strong hydrophobic contact with the Rutin ring at 48 % of the total simulation trajectory. The hydroxyl group of mannopyranosyl moiety formed weak hydrogen bonding interactions with Ile472 and Thr470 at 21 % and 14 % of the simulation trajectory. The RMSD, radius of gyration, molecular surface area MSA, SASA, PSA of rutoside (Supplementary Figure S7) interacting in the catalytic pocket of *6YZ5.pdb* were observed to be 3.3–4.3 Å, 4.4–4.9 Å, 422.26–480.04 2 Å, 390.70–570.132 Å, and 425.65–499.872 Å respectively.

#### 3.4.3 Molecular Dynamics Simulation Study of Rutoside/7BTF Complex

**Figure 8:**
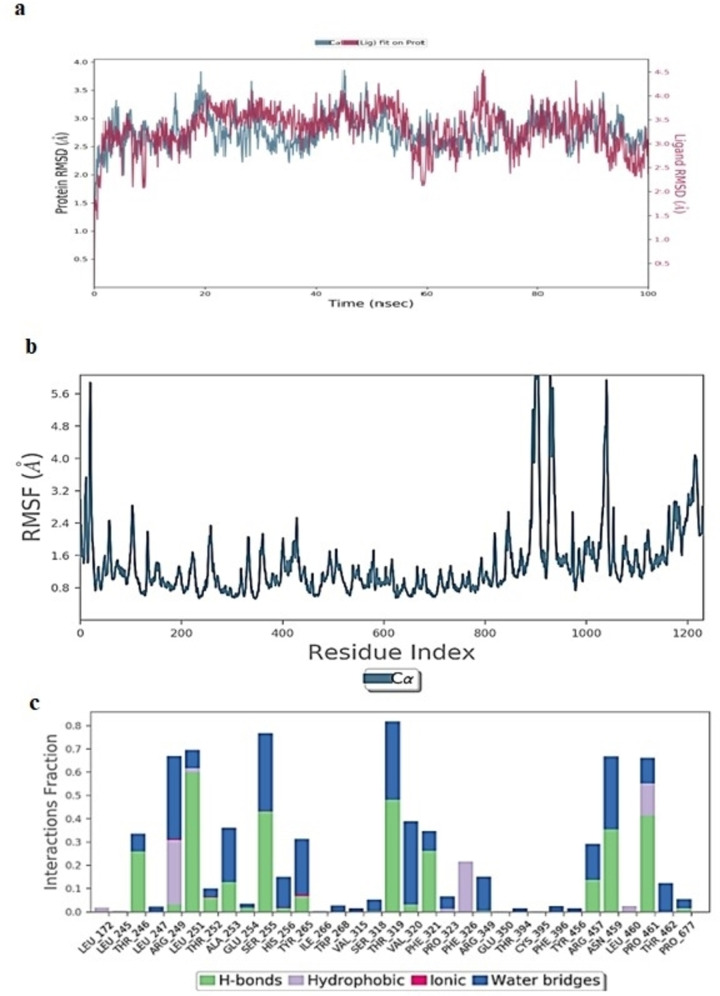
(A) RMSD (Å) of simulated protein 7BTF.*pdb* in complex with inhibitor rutosideover 100 ns MD simulation (B) RMSF of simulated protein 7BTF.*pdb* in complex with inhibitor rutoside over 100 ns MD simulation (C) Interaction fraction of 7BTF.*pdb* complexed with rutosideover 100 ns MD simulation.

The docked pose of rutoside/7BTF was simulated at 100 ns by placing it in an orthorhombic box solvated with TIP3P waters and the system was neutralized by adding Na^+^ and Cl^−^ ions. The total system consists of 150200 atoms and 43471 water molecules. The 100 ns MD trajectory of the simulated rutoside/7BTF complex revealed that the RMSD of protein Cα and backbone atoms was observed in the range of 1.95 to 3.22 Å and 1.96 to 3.28 Å respectively (Figure 8A). From Figure 10 ligand was equilibrated at 34 ns from which it is stabilized until a total 100 ns simulation time with RMSD of 2.2–3.2 Å. The RMSF (root mean square fluctuation) plot as represented in Figure 8B indicated that the protein is stable with 0.9–2.0 Å, except for amino acid residues from 900 showing major fluctuations.

The monitored protein interaction fraction plot indicated that the majority of hydrogen bonding interactions stabilized rutoside in the catalytic pocket of 7BTF. Hydrophobic, ionic, and water‐bridged interactions played a minor role in stabilizing rutoside. Thr246, Lue251, Thr252, Ala253, Ser255, Thr319, Phe321, Arg457 and Pro461 interacted with rutoside over 10–60 % of 100 ns simulation time (Figure 8C). The timeline representation of protein‐ligand contacts is outlined in Supplementary Figure S8. The 2D ligand interaction diagram (Supplementary Figure S9) illustrated that, the multiple hydroxyl groups of rutoside were stabilized by forming nine hydrogen bonds with catalytic residues. The two hydroxyl groups of 3,4‐dihydroxy phenyl ring formed low to medium‐range multiple bidentate hydrogen bonds with Ser255 and Lue251 at 16–32 % of the simulation trajectory. The hydroxyl group at 7^th^ position of the aromatic ring fused with pyran ring accepted electrons from the backbone and side chain atoms of Pro461 and Thr246 with 41 and 22 % of simulation trajectory. The same ring interacted with Arg249 in a π‐cation hydrophobic manner. The hydroxyl groups of mannopyranosyl glycoside moiety interacted forming low fraction hydrogen bonds with Phe321 and Asn459. One low fraction water‐bridged interaction was observed with the oxygen of the mannopyranose ring. The properties of rutoside (Supplementary Figure S10) interacting in the catalytic pocket of 7BTF.*pdb* like RMSD, the radius of gyration, molecular surface area (MSA), solvent accessible surface area (SASA), and polar surface area (PSA) of ligand was observed in the range of 0.8–2.7 Å, 4.5–4.8 Å, 450.19–485.24 Å^2^, 180.23–280.11 Å^2^ and 440.25–490.37 Å^2^ respectively. These properties inferred major fluctuations after 34 ns following stabilization throughout the end of simulation may be due to loss of contacts between residues Phe321 and Asn459 with hydroxyl groups of glycoside moiety.

From the earlier studies where through molecular docking, molecular dynamics, and *in vitro* studies it was elucidated that rutin and flavone analogs displayed inhibitory activity against the Mpro of SARS ‐CoV‐2.

#### 3.4.4 Molecular Dynamics Simulation Study of Erioicitrin

**Figure 9:**
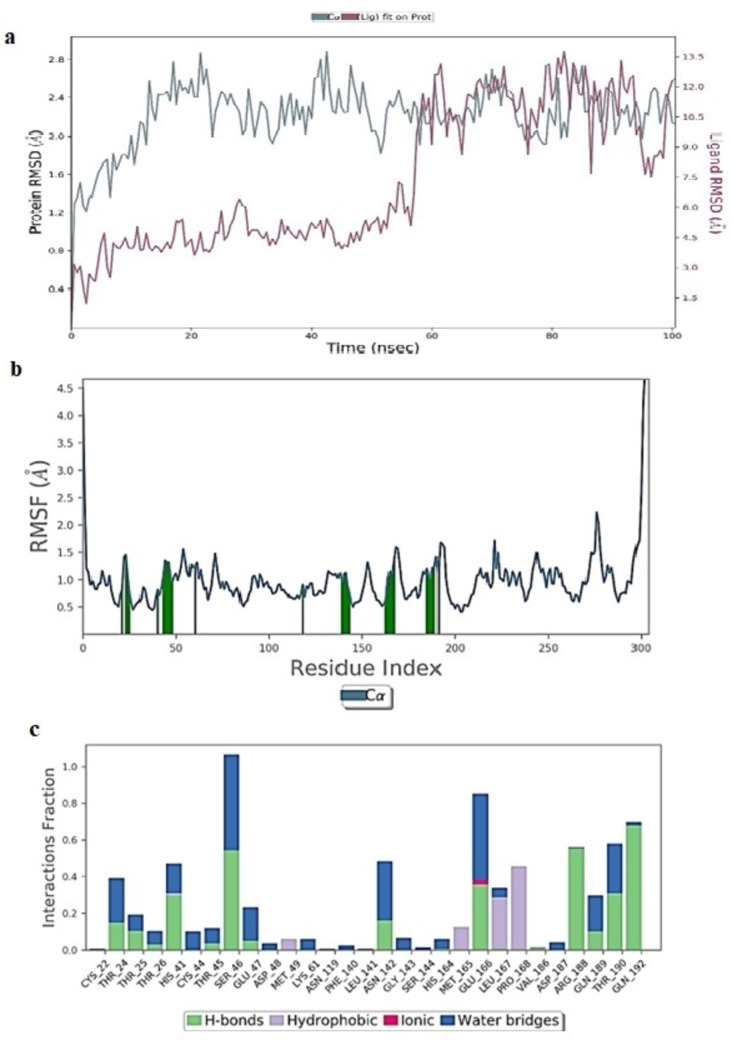
(A) RMSD (Å) of simulated protein 5R82.*pdb* in complex with inhibitor eriocitrinover 100 ns MD simulation (B) RMSF of simulated protein 5R82.*pdb* in complex with inhibitor eriocitrin over 100 ns MD simulation (C) Interaction fraction of 5R82.*pdb* complexed with eriocitrinover 100 ns MD simulation.

The RMSD for the Cα atoms oferiocitrin/Mpro complex exhibited fluctuations ranging from 2.01–2.78 Å, whereas RMSD of eriocitrin ligand showed major fluctuations at 60 ns and later ranged from 1.85–2.85 Å throughout 100 ns simulation (Figure 9A). The RMSF values (Figure 9B) of amino acids were low reaching 1.5–2.2 Å implying protein stability. From Figure 9C, eriocitrin formed the majority of hydrogen bonds and water‐bridged interactions with a low frequency of hydrophobic bonds.

**Figure 10:**
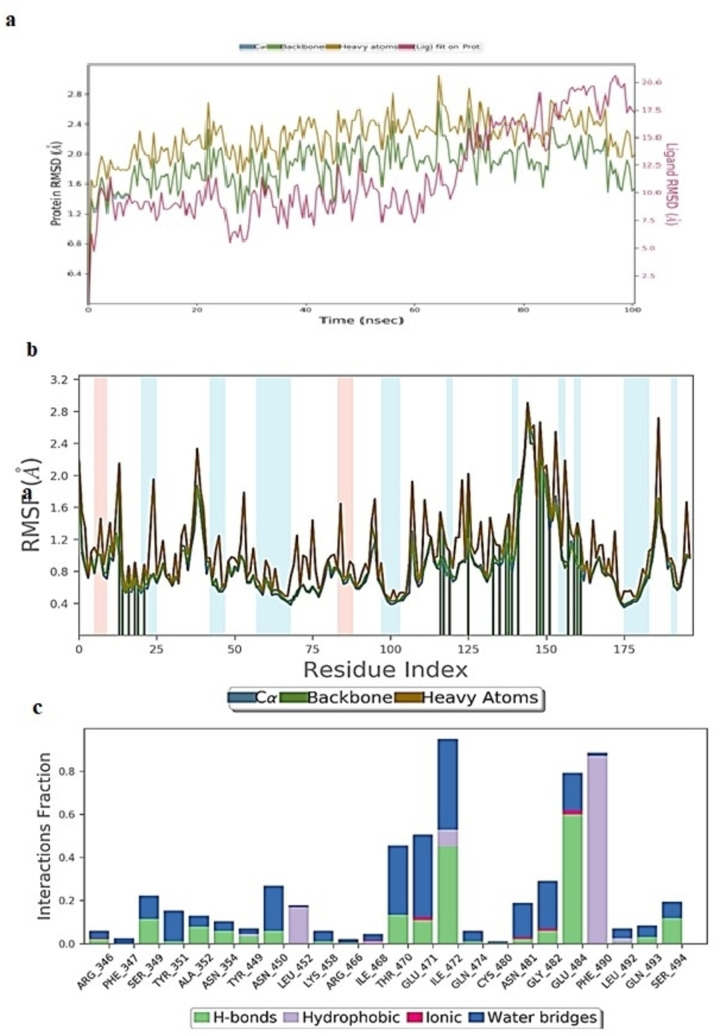
(A) RMSD (Å) of simulated protein 6YZ5.*pdb* in complex with inhibitor eriocitrinover 100 ns MD simulation (B) RMSF of simulated protein 6YZ5.*pdb* in complex with inhibitor eriocitrin over 100 ns MD simulation (C) Interaction fraction of 6YZ5.*pdb* complexed with reriocitrinover 100 ns MD simulation.

Whereas in the eriocitrin/spike glycoprotein complex (Figure 10A) the Cα and backbone atoms showed minor fluctuations in RMSD ranging from 1.24–2.58 Å with minor spike in RMSD after 60 ns. The ligand RMSD (Figure 10A) hiked after 70 ns reaching 20 Å at the end of the 100 ns simulation, indicating unstable binding of eriocitrin with spike glycoprotein. The swift changes in the binding pattern of eriocitrin after 70 ns is due to the orientation of molecules forming new hydrogen bonds with Ser349, Asn450 and Leu452.From Figure 10B, protein RMSF exhibited higher values indicating poor bonding of eriocitrin with catalytic residues. This was further confirmed by the protein‐ligand interaction plot (Figure 10C) by the formation of low‐fraction hydrogen bonds with catalytic pocket amino acids.

**Figure 11:**
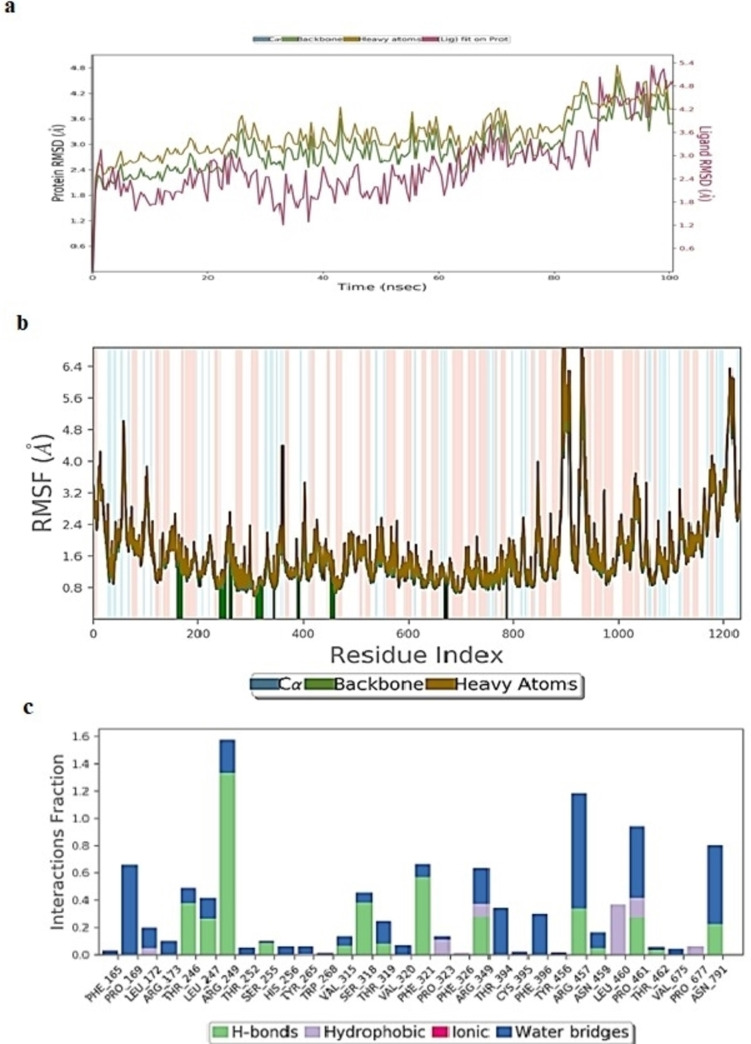
(A) RMSD (Å) of simulated protein 7BTF.*pdb* in complex with inhibitor Eriocitrin over 100 ns MD simulation (B) RMSF of simulated protein 7BTF.*pdb* in complex with inhibitor eriocitrin over 100 ns MD simulation (C) Interaction fraction of 7BTF.*pdb* complexed with eriocitrinover 100 ns MD simulation.

The RMSD of Cα, backbone and ligand atoms in RdRpwhen complexed with eriocitrin exhibited gradual incline by the end of 100 ns simulation (Figure 11A). The RMSF values (Figure 11B) of the ligand‐bound amino acids were found low reaching 1.62 Å.From Figure 11C, the ligand was stabilized in the active pocket through the formation of hydrogen bonds, where amino acids Arg249, Arg457, Asn791, and Pro461 formed a major fraction of hydrogen bonding.

Comparing the conformational dynamic changes of rutoside and eriocitrinin the catalytic pockets of three proteins Mpro, Spike glycoprotein, and RdRp figured out that the structural changes in both flavones altered the degree of interactions with catalytic residues.

#### *3.4.5. In Vitro* Assay of 3CL Protease or Mpro (B.1.1.529, Omicron Variant, P132H mutant) SARS‐CoV‐2

**Figure 12:**
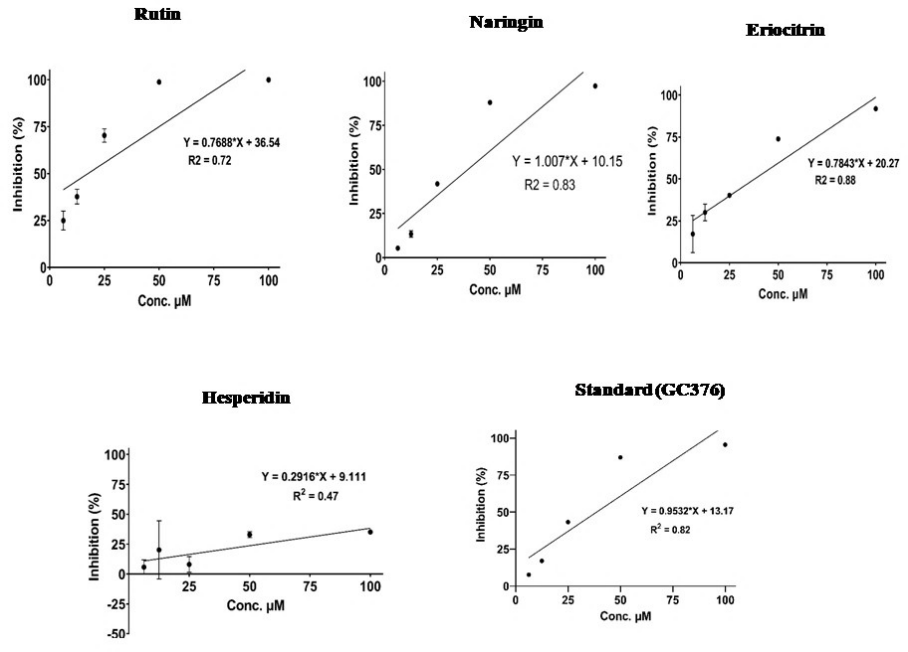
Fluorescent intensity of 3CL protease or (Mpro) (B.1.1.529, Omicron variant, P132H mutant) SARS‐CoV‐2.

We performed assay with significant Natural compounds from *in silico* results (Rutin, Eriocitrin, Naringin, Hesperidine) using 3CL protease assay kit (B.11529 Omicron variant). SARS‐CoV‐2, 3CL protease (B.1.1.529, Omicron Variant) (SARS‐CoV‐2) Assay Kit, BPS Bioscience, USA. was purchased through Inveniolife Technology Pvt. Ltd., Office# 118, Grow More Tower, Plot# 5, Sector‐2, Kharghar, Navi Mumbai – 410210, India [Suplimentary Purchase Bill Voucher (V1)].This kit contained 3CL inhibitor GC376 as Control. The IC_50_ value of the test compound was found to be Rutin −17.50 μM, Eriocitrin‐ 37.91 μM, Naringin −39.58 μM, Hesperidine −140.20 μM, the standard inhibitory concentration of GC376 was 38.64 μM (Figure 12 **)**. These findings suggest that Rutin, Eriocitrin and Naringin showed maximum activity, but hesperidine showed less promising activity as compared to standard (GC376) (Table 3).[^57^, ^58^, ^59^, ^60^, ^61^]

**Table 3:** IC_50_ values of Test and Standard compounds.

Compound	IC_50_ (μM)
Rutin	17.50
Eriocitrin	37.91
Naringin	39.58
Hesperidine	140.20
GC376 (Std)	38.64

## 4 Conclusions

In conclusion, the binding affinities of major chemical constituents of *C. limon* against multi targets of SARS‐CoV‐2 such as Mpro (5R82.*pdb*), Spike glycoprotein (6YZ5.*pdb*), and RNA‐dependent RNA polymerase (RdRp) (7BTF.*pdb*) for COVID‐19 are more significant by *in silico* methods using Schrodinger suite. From the molecular docking and molecular dynamic studies, the chemical constituents from lemon showed significant binding energy against various targets of SARS‐Cov‐2 when compared to standard drugs. The 100 ns MD simulation of the docked pose of rutoside/5R82, rutoside/6YZ5, rutoside/7BTF, eriocitrin/5R82, eriocitrin/6YZ5 and eriocitrin/7BTF complex revealed that these two compounds formed moderate to strong hydrogen bonds with the catalytic residues. Results from these studies notify that the compounds Rutoside, Naringin, Eriocitrin, Hesperidin, and Quercetin from lemon may synergize remedially in combination with anti‐SARS‐CoV‐2 agents. From the current study, the binding pattern of *C. limon* chemical constituents against three targets of SARS‐CoV‐2 Mpro, Spike protein, and RNA‐dependent RNA polymerase (RdRp) provided preliminary insights suggesting the mechanistic approach in further design of new therapeutic agents for the treatment of COVID‐19. In conclusion, our results indicate that Flavionoids – (Rutin, Eriocitrin, Naringin, Hesperidine) can efficiently inhibit SARS‐CoV‐2 infection. *In vitro* study for 3CL protease or Mpro (B.1.1.529, Omicron variant, P132H mutant) was performed by SARS‐CoV‐2 assay. We predicted that the drugs have a good potential to combat the disease and can be taken as a good candidate for further clinical trials. Also, the immunomodulatory activity of flavonoids from *C. limon* may help in boosting immunity that helps the prevention of COVID‐19.[^62^, ^63^, ^64^, ^65^, ^66^]

## Author Contributions

Conceptualization, writing the original draft, formal analysis: Kannan Raman, Rajagopa Kalirajan, Fahadul Islam, Srikanth Jupudi, Divakar Selvaraj, Gomathi Swaminathan, Laliteshwar Pratap Singh. Investigations, funding acquisition, resources, project administration, reviewing and editing: Shopnil Akash, Md. Rezaul Islam, Ritesh Rana, Firzan Nainu. Data validation, and reviewing and editing: Talha Bin Emran, Turki M. Dawoud, Mohammed bourhia, and Rashu Barua.

## Conflict of Interests

The authors declare no conflict of interest.

## Supporting information

As a service to our authors and readers, this journal provides supporting information supplied by the authors. Such materials are peer reviewed and may be re‐organized for online delivery, but are not copy‐edited or typeset. Technical support issues arising from supporting information (other than missing files) should be addressed to the authors.

Supporting Information
