# Supplementary material for: Phytoconstituents of Citrus limon (Lemon) as Potential Inhibitors Against Multi Targets of SARS‐CoV‐2 by Use of Molecular Modelling and In Vitro Determination Approaches
Source: ChemistryOpen. 2024 Jun 21;13(10):e202300198. doi: 10.1002/open.202300198 (PMC11457754; doi:10.1002/open.202300198)

ChemistryOpen

Supporting Information

Phytoconstituents of *Citrus limon* (Lemon) as Potential Inhibitors Against Multi Targets of SARS-CoV-2 by Use of Molecular Modelling and *In Vitro* Determination Approaches

Kannan Raman, Rajagopal Kalirajan,* Fahadul Islam, Srikanth Jupudi, Divakar Selvaraj, Gomathi Swaminathan, Laliteshwar Pratap Singh, Ritesh Rana, Shopnil Akash, Md. Rezaul Islam, Firzan Nainu, Talha Bin Emran, Turki M. Dawoud, Mohammed Bourhia,* Musaab Dauelbait, and Rashu Barua

Research Article

Phytoconstituents of *Citrus limon* (lemon) as potential inhibitors against multi targets of SARS-CoV-2 by Molecular Modelling approach and *in vitro* determination against Mpro (Omicron variant - B.1.529)

Supplemenatry Material

Table-S1 *In silico* ADMET screening for Phytoconstituents of *Citrus limon*

Compound	Mol. Wt.	Dipole	Donor HB	Accpt HB	QPlog o/w	#metab	Rule of Five	%Human Oral Absorption
L1_Citric acid	192.125	6.801	3	5.75	0.045	3	0	16.37
L2_Limonene	136.236	0.306	0	0	3.981	5	0	100
L3_Terpinene	136.236	0.048	0	0	4.051	4	0	100
L4_Alpha Terpeneol	154.252	1.661	1	0.75	2.95	4	0	100
L5_4_Terpeneol	154.252	1.974	1	0.75	2.961	4	0	100
L6_Geraniol	154.252	2.151	1	1.7	2.613	6	0	100
L7_Beta Ocimene	136.236	0.228	0	0	4.335	4	0	100
L8_Linalool	154.252	1.994	1	0.75	3.104	4	0	100
L9_Apigenin	270.241	3.316	2	3.75	1.616	3	0	73.33
L10_Limocitrin	346.293	4.379	3	6	1.38	6	0	68.26
L11_Quercetin	302.24	4.838	4	5.25	0.349	5	0	52.25
L12_Eriocitrin	594.525	8.32	8	19.8	-1.958	9	3	20.32
L13_Hesperidine	606.579	7.594	7	18.1	-0.492	9	3	22.25
L14_Naringin	578.526	4.976	7	19.05	-1.479	8	3	21.31
L15_Rutoside	610.524	8.98	9	20.55	-2.344	10	3	20.78
Hydroxychloroquine(std)	335.876	6.854	2	5.7	3.369	5	0	93.21
**Recommended values**	130-725	1-12.5	0– 6	2-20	-2-6.5	1 – 8	max 4	>80% is high <25% is poor

MW- Molecular weight of the molecule,
Dipole – Computed dipole moment
donorHB - Estimated number of hydrogen bonds that would be donated by the solute to water molecules in an aqueous solution.
accptHB- Estimated number of hydrogen bonds that would be accepted by the solute from water molecules in an aqueous solution
QPlogPo/w - Predicted octanol/water partition coefficient.
#metab- Number of likely metabolic reactions.
RuleOfFiveNumber of violations of Lipinski's rule of five.
%Human- Oral absorption- Predicted human oral absorption on 0 to 100% scale.

Fig-S1a Docking of compounds L1-L15 with SARS CoV-2 main protease (5R82)

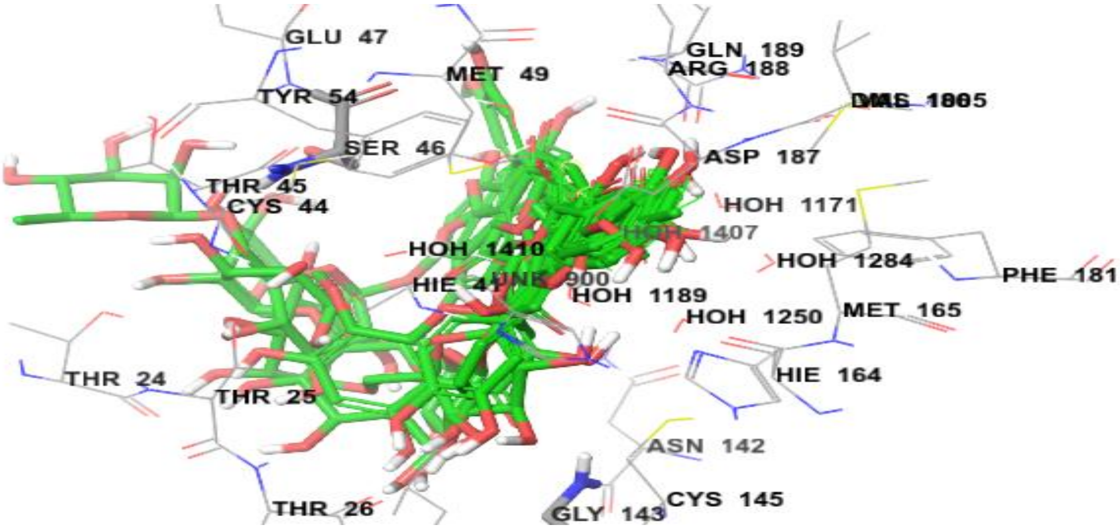

Fig- S1b Docking of compounds L1-L15 with SARS CoV-2 Spike protein (6YZ5)

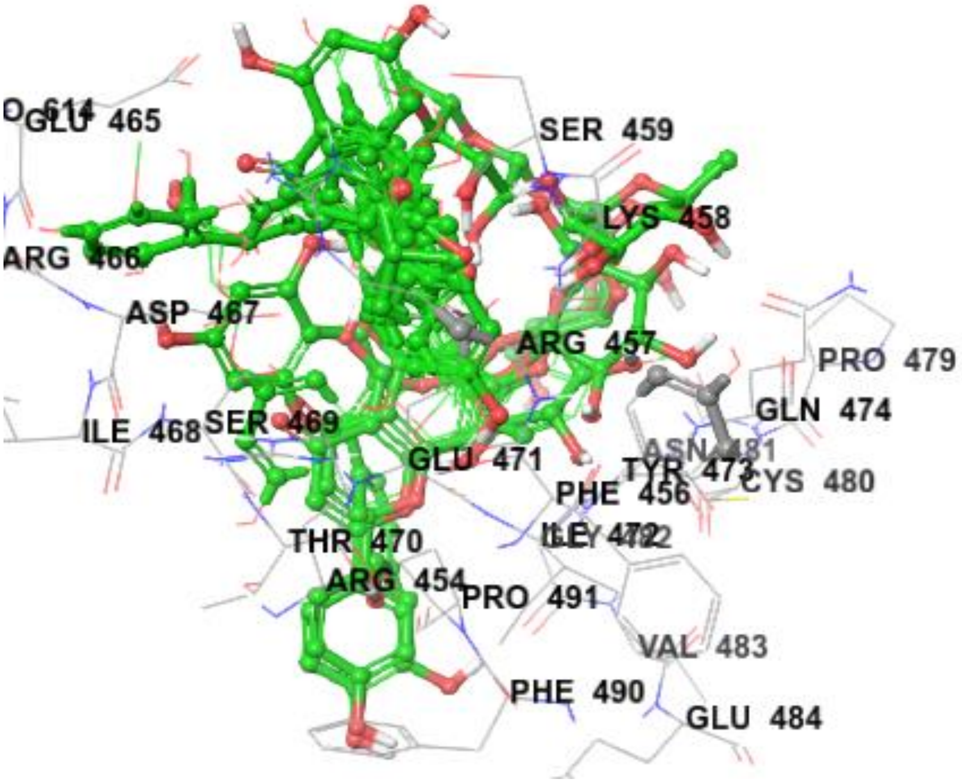

Fig- S1c Docking of compounds L1-L15 with SARS CoV-2 RdRp (7BTF)

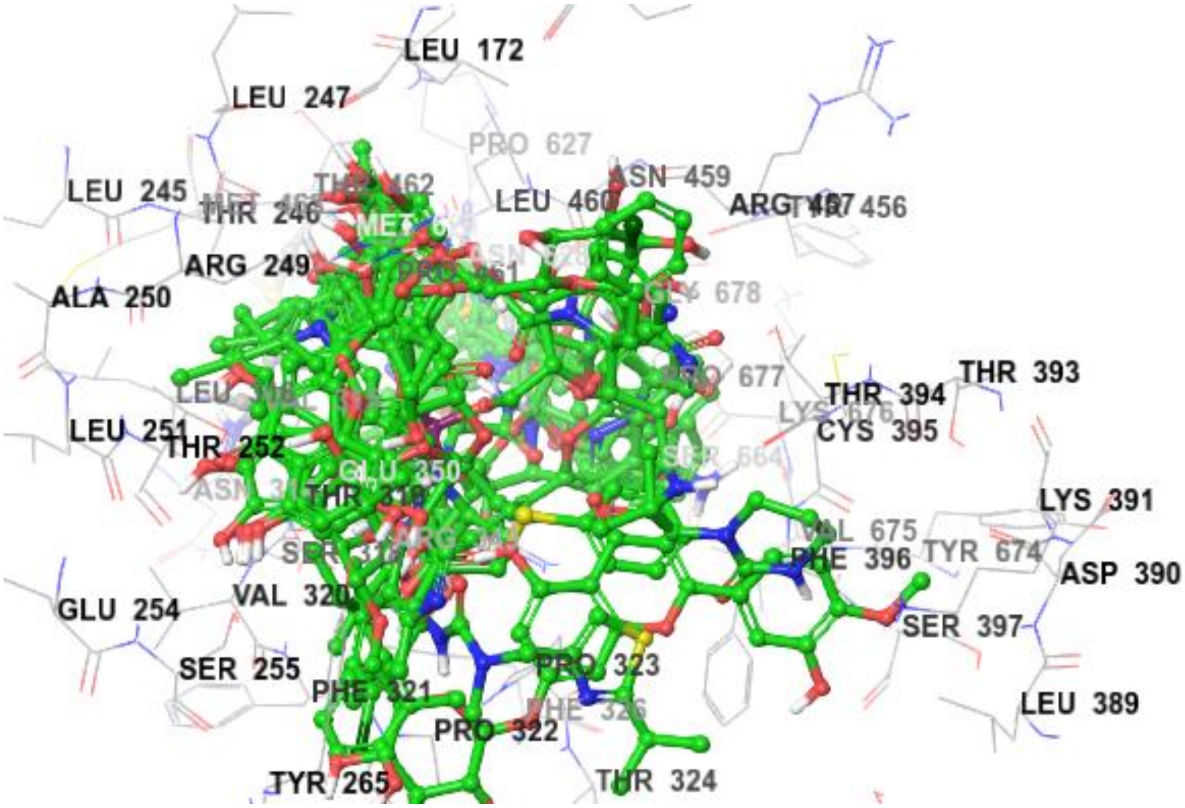

Figure S2: Time line representation showing different contacts formed by Rutoside in complex with 5R82.*pdb* during 100 ns MD simulation.

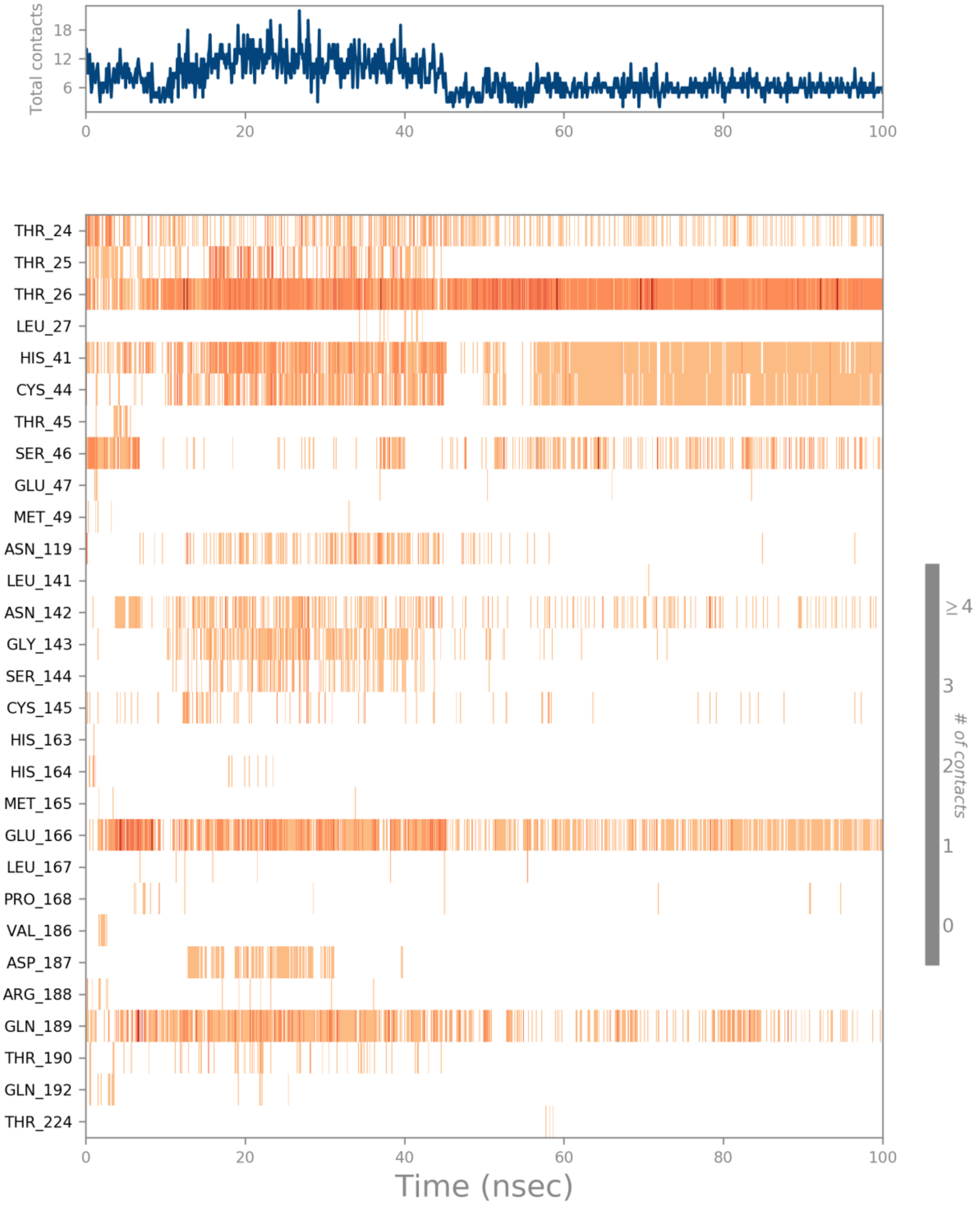

Figure S3: 2D interaction diagram of Rutoside in complex with 5R82.*pdb* during 100 ns MD simulation

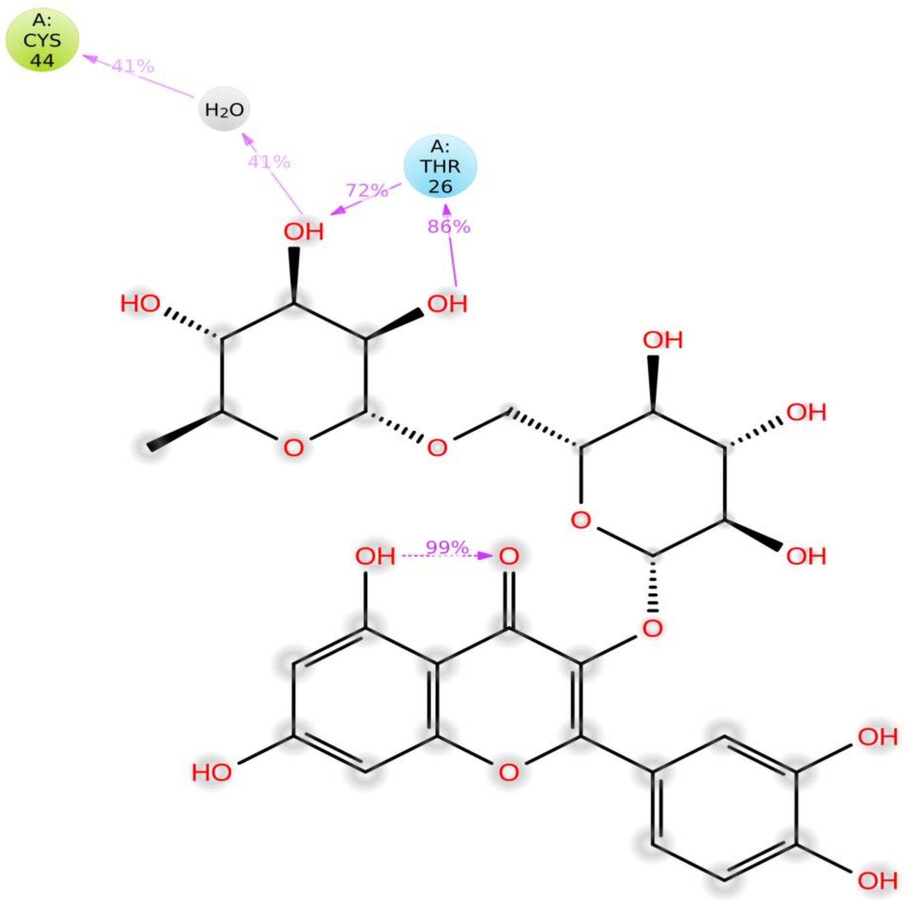

Figure S4: Ligand properties of Rutoside in complex with 5R82.*pdb* during 100ns MD simulation.

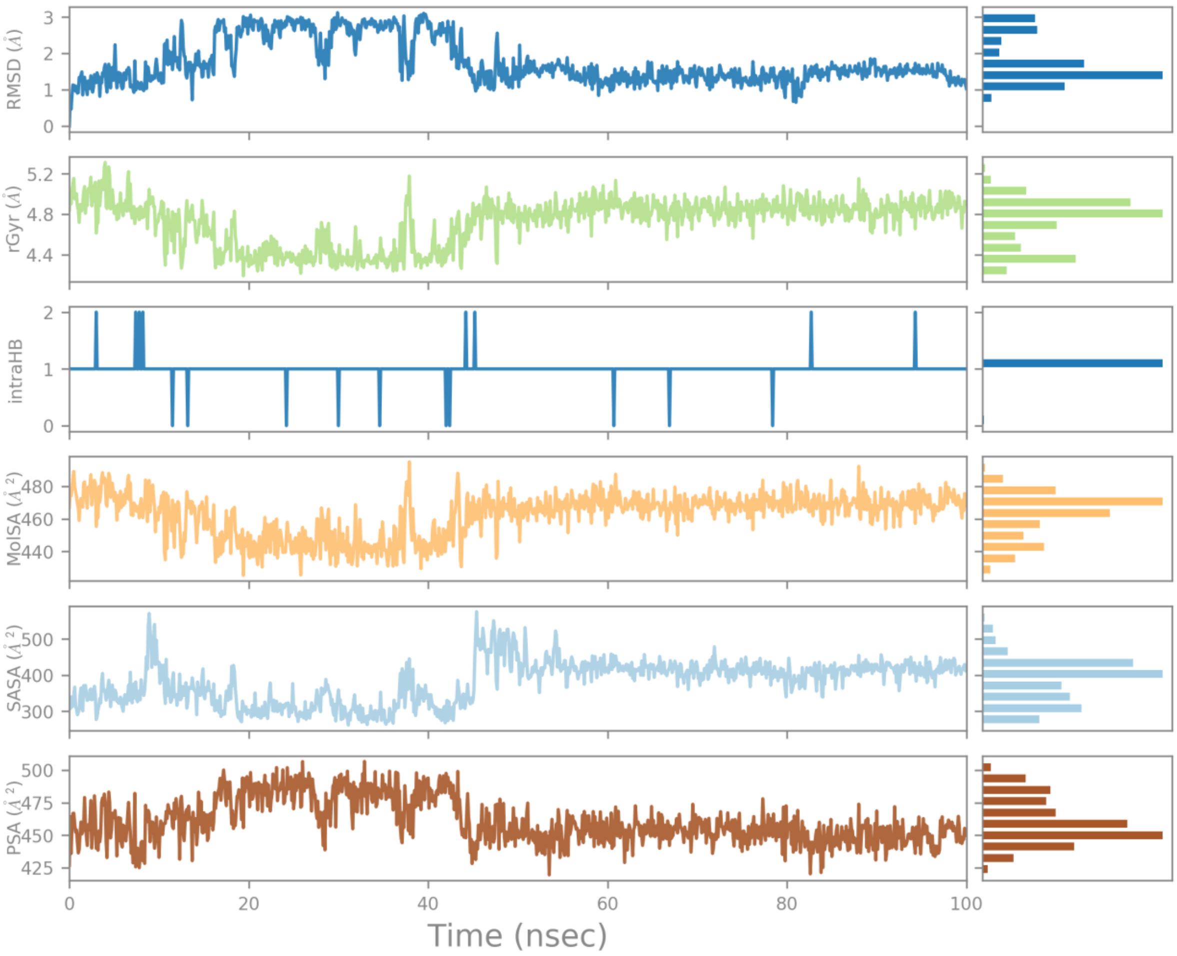

Figure S5: Time line representation showing different contacts formed by Rutoside in complex with 6YZ5.*pdb* during 100 ns MD simulation.

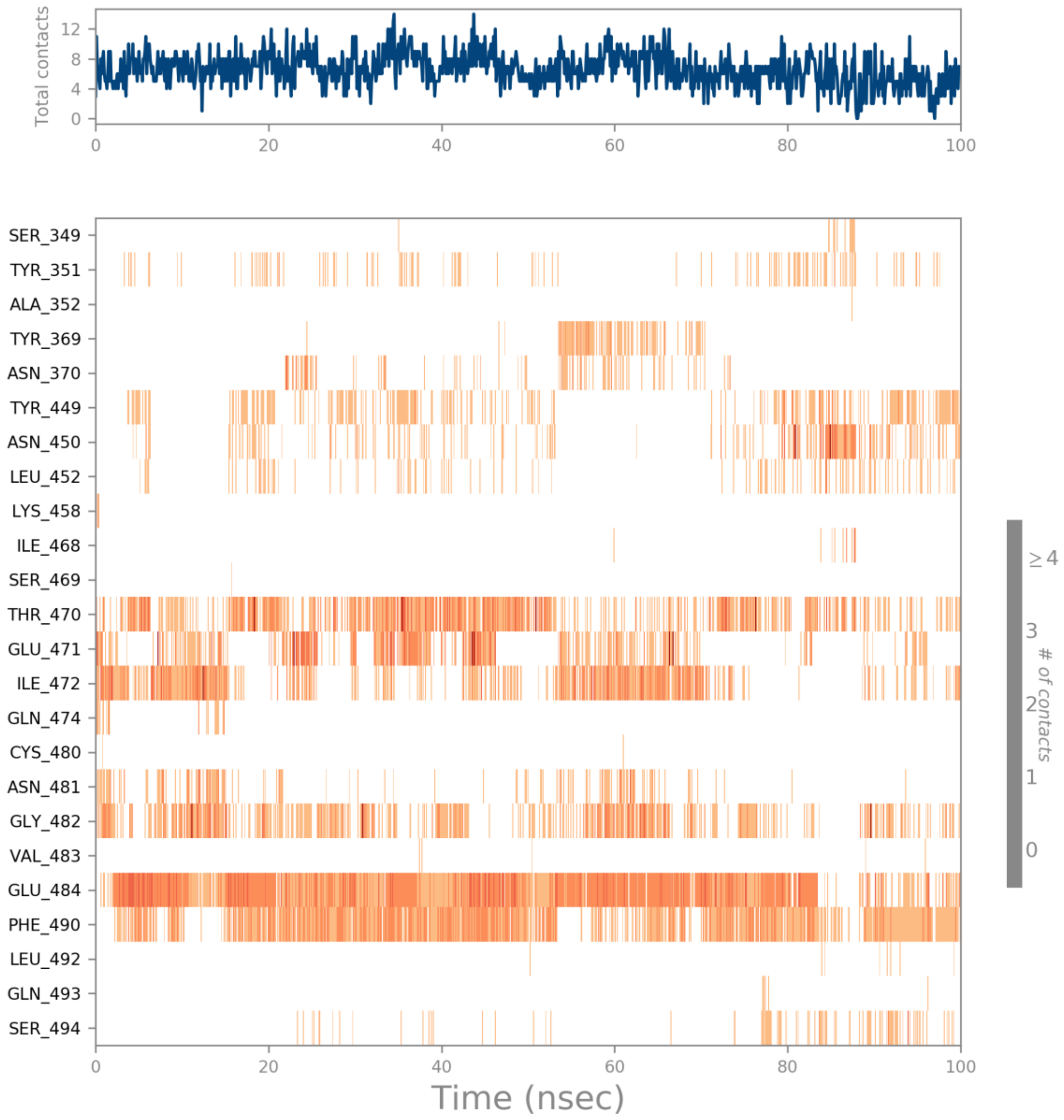

Figure S6: 2D interaction diagram of Rutoside in complex with 5R82.*pdb* during 100 ns MD simulation

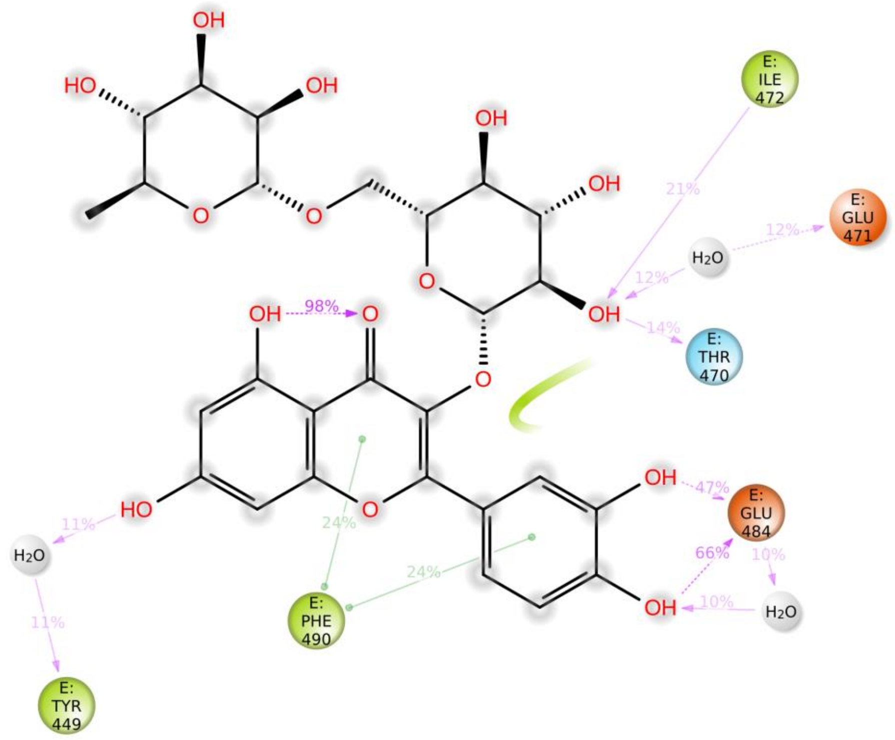

Figure S7: Ligand properties of Rutoside in complex with 6YZ5.*pdb* during 100ns MD simulation.

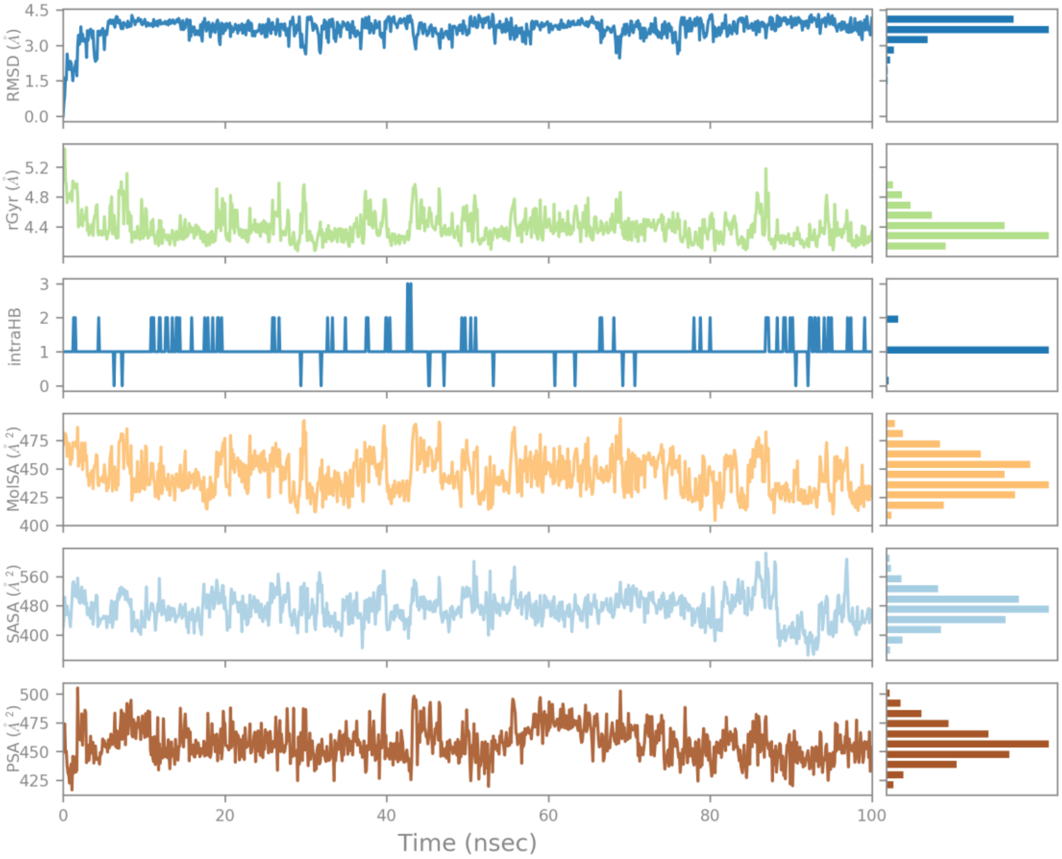

Figure S8: Time line representation showing different contacts formed by Rutoside in complex with 7BTF.*pdb* during 100 ns MD simulation.

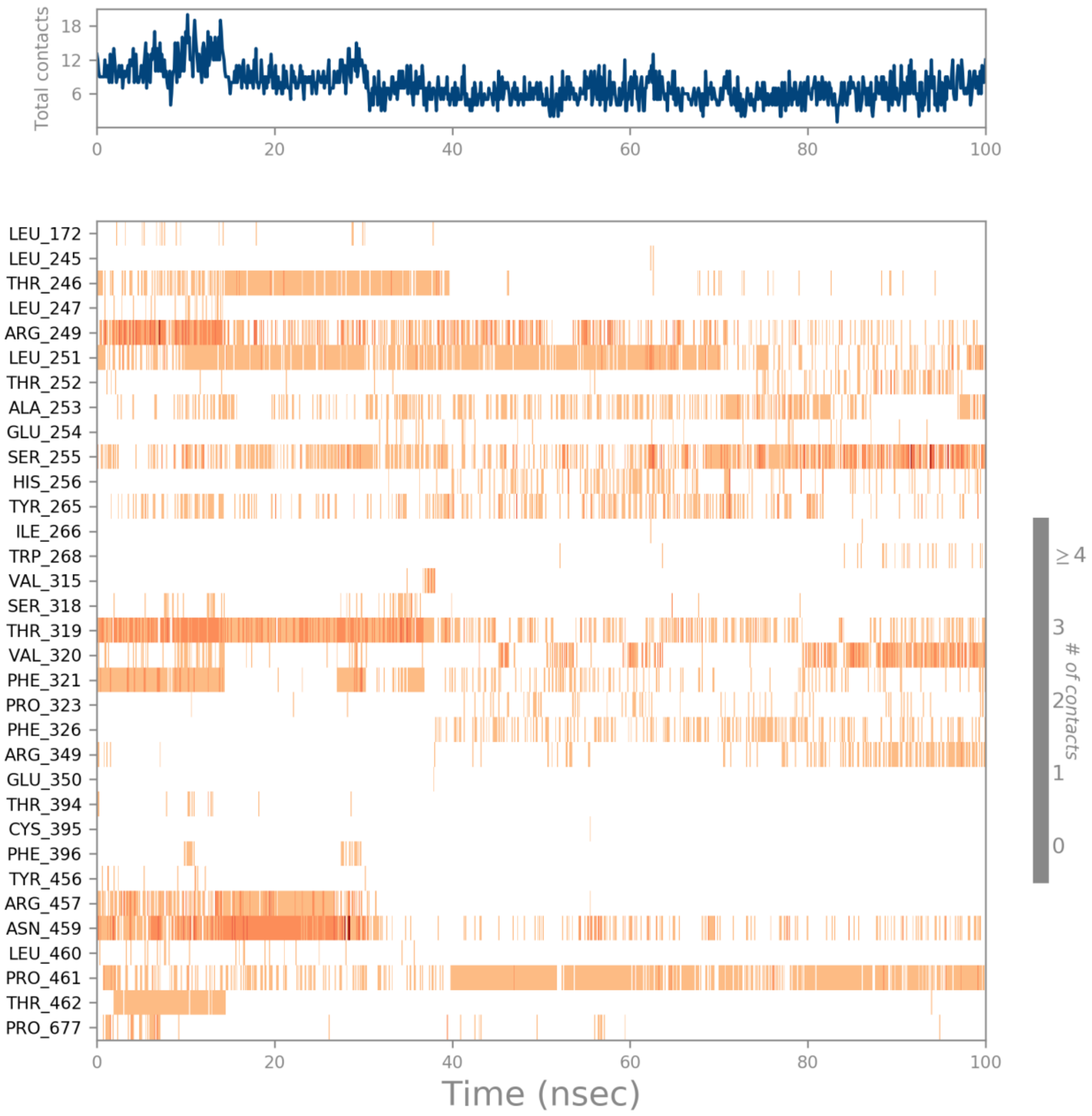

Figure S9: 2D interaction diagram of Rutoside in complex with 7BTF.*pdb* during 100 ns MD simulation

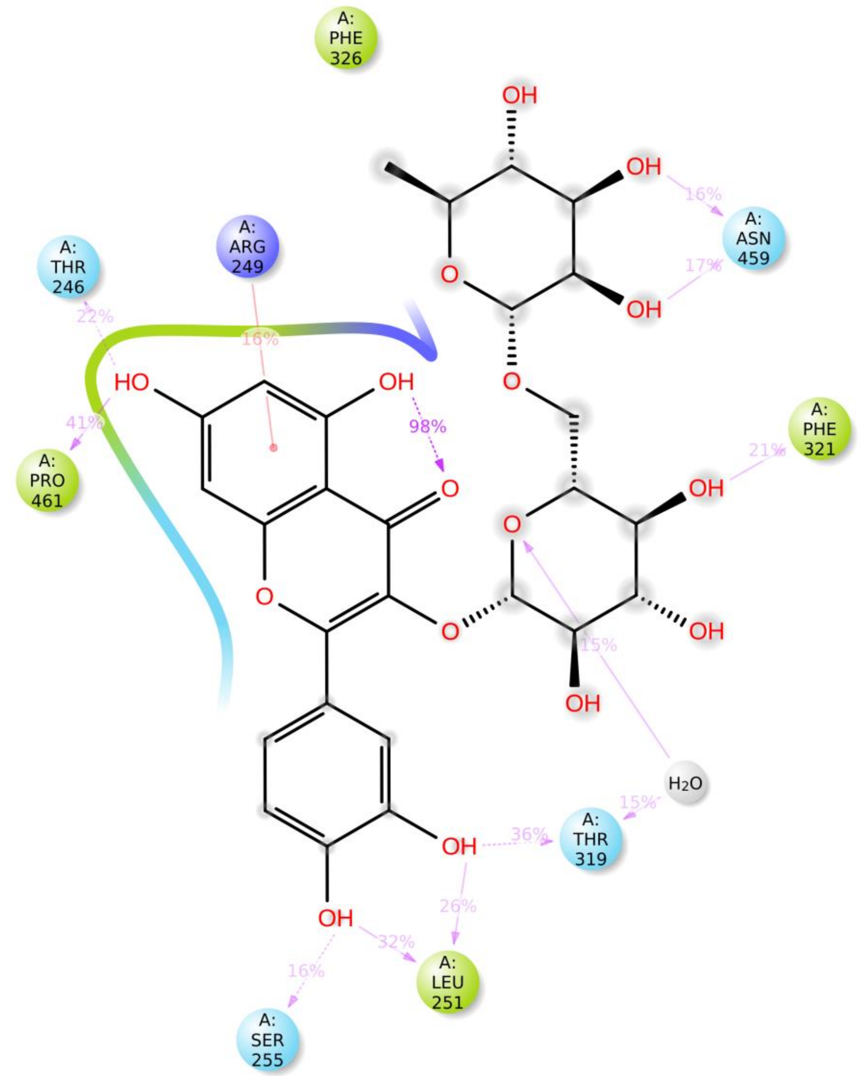

Figure S10: Ligand properties of Rutoside in complex with 7BTF.*pdb* during 100ns MD simulation.

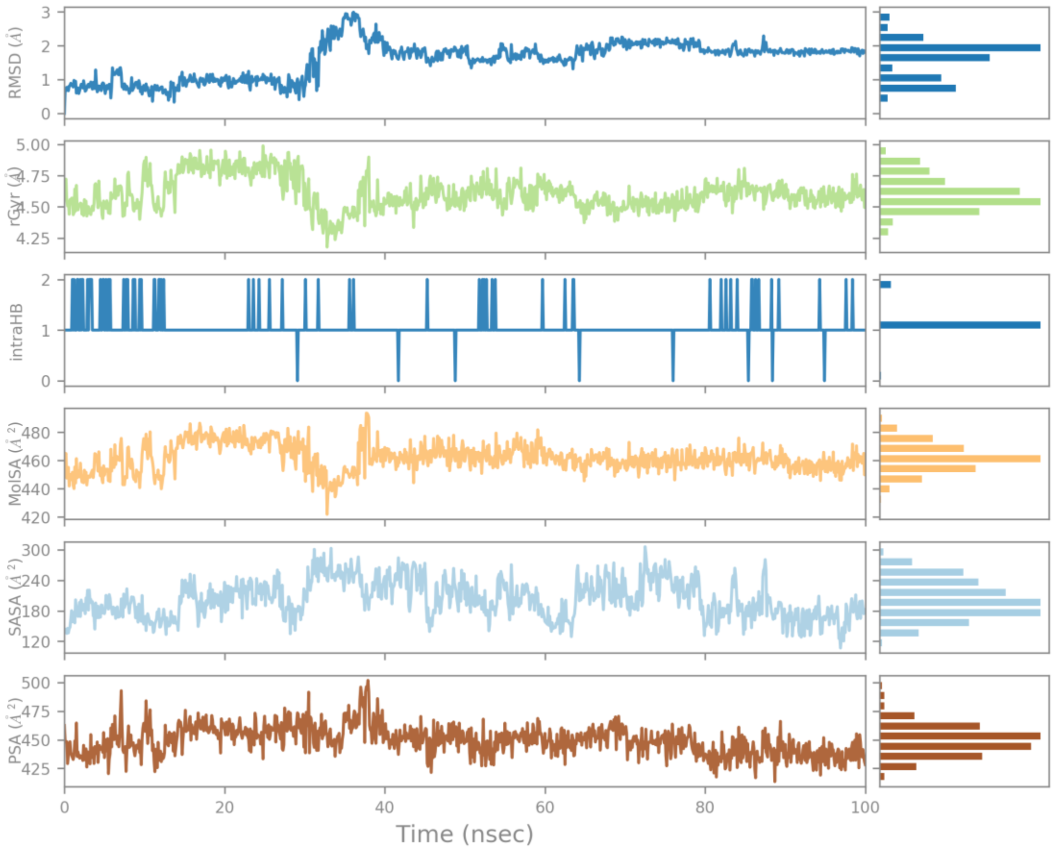

Supplement: Supplementary file 1 — Supporting Information [file OPEN-13-e202300198-s001.pdf]
